# Type 2 diabetes and cause-specific mortality in Mexico City: a Mendelian randomisation analysis

**DOI:** 10.1016/j.lana.2025.101082

**Published:** 2025-04-06

**Authors:** Fiona Bragg, Pablo Kuri-Morales, Eirini Trichia, Jason M. Torres, Paulina Baca, Adrián Garcilazo-Ávila, Carlos González-Carballo, Raul Ramirez-Reyes, Fernando Rivas, Diego Aguilar-Ramirez, Louisa Gnatiuc-Friedrichs, William G. Herrington, Michael Hill, Tianshu Liu, Alejandra Vergara, Rachel Wade, Rory Collins, Richard Peto, Jaime Berumen, Jesus Alegre-Díaz, Jonathan R. Emberson, Roberto Tapia-Conyer

**Affiliations:** a Clinical Trial Service Unit & Epidemiological Studies Unit, Nuffield Department of Population Health, University of Oxford, Oxford, UK; b Health Data Research UK Oxford, University of Oxford, Oxford, UK; c Faculty of Medicine, National Autonomous University of Mexico, Mexico City, Mexico; d Instituto Tecnológico y de Estudios Superiores de Monterrey, Monterrey, Mexico; e Experimental Research Unit from the Faculty of Medicine, National Autonomous University of Mexico, Mexico City, Mexico

**Keywords:** Mendelian randomisation, Mexico, Mortality, Type 2 diabetes

## Abstract

**Background:**

Observational epidemiological studies in Mexico have shown high mortality risks associated with type 2 diabetes (T2D). However, it is unclear whether these relationships are wholly causal. We aimed to assess the association of genetically-predicted T2D liability with risk of death in Mexico.

**Methods:**

Between 1998 and 2004, 150,000 men and women were recruited from Mexico City and followed-up until September 2022 for cause-specific mortality. Mendelian randomisation analyses, using a genetic risk score (GRS) comprising 1055 established T2D-associated risk variants, estimated associations with risk of all-cause and cause-specific mortality at ages 35–74.

**Findings:**

Among 121,433 included participants with a mean (standard deviation) age of 51 (11), 68% (n = 82,249) were women and 18% (n = 21,371) had T2D. The GRS explained 6.3% of T2D liability and was not associated with major potential confounders of the T2D-mortality relationship. During a median (interquartile range) of 20.2 (19.4–21.4) years’ follow-up, 12,293 participants died. Genetically-predicted T2D liability was associated with a death rate ratio (RR) of 1.29 (95% confidence interval [CI] 1.23–1.36) per trebling in genetically-predicted odds of T2D. There were particularly strong associations with death from renal disease (n = 1696; RR 2.29 [95% CI 1.99–2.64]) and acute diabetic crises (n = 509; RR 2.27 [1.75–2.93]) and weaker, but still strong, associations with death from vascular disease (n = 3226; RR 1.31 [1.19–1.46]) and infection (n = 2437; RR 1.21 [1.07–1.36]). Genetically-predicted T2D liability was not clearly associated with death from cancer (n = 2016; RR 1.00 [95% CI 0.88–1.14]) or cirrhosis (n = 895; RR 0.90 [0.74–1.10]).

**Interpretation:**

T2D is causally associated with death from vascular, renal and infectious diseases. Its prevention and effective management could substantially reduce premature deaths in Mexico, where T2D is common.

**Funding:**

10.13039/100010269Wellcome Trust, the Mexican Health Ministry, the 10.13039/501100003141National Council for Science and Technology (CONACyT) for Mexico, Cancer Research UK, 10.13039/501100000274British Heart Foundation, Kidney Research UK, UK 10.13039/501100000265Medical Research Council, 10.13039/100004325AstraZeneca, Regeneron.

**Research in context**

**Evidence before this study**

We searched PubMed for articles published in English prior to November 4, 2024, using the search terms (“Mendelian randomisation” OR “genetic instrument” OR “instrumental variable” OR “genetic risk score”) AND “type 2 diabetes”. Reference lists of relevant articles were reviewed. Multiple Mendelian randomisation studies exploring disease associations of genetically-predicted type 2 diabetes (T2D) were identified. These found positive associations of genetically-predicted T2D liability with renal and cardiovascular diseases, consistent with causal relationships, but findings for other diseases were inconsistent. Most identified studies employed a two-sample Mendelian randomisation approach, using summary statistics from European ancestry genome-wide association studies, and none was undertaken in a Mexican population. Few studies included comparison of genetic associations of T2D with observational associations, and most examined single diseases or groups of closely related diseases.

**Added value of this study**

The associations of genetically-predicted T2D liability in the present study population, comprising approximately 150,000 men and women from Mexico City, were consistent with causal relationships of T2D with premature death from vascular, renal and infectious diseases. These findings corroborate those of previous studies demonstrating causal associations of T2D with cardiovascular and renal diseases. However, prior evidence regarding the causal relevance of T2D for infectious diseases has been inconsistent. The presented findings from the Mexico City Prospective Study therefore provide valuable new evidence supporting a causal role for T2D in death from infection. Moreover, the associations of genetically-predicted T2D liability in the present study appear stronger than previously reported causal effects of T2D on renal, vascular and infectious diseases in European and East Asian ancestry populations. This suggests the stronger T2D-associated mortality risks observed among adults in Mexico likely reflect, at least in part, strong causal effects of T2D in this population, and not just the effects of residual confounding or biases. There remains uncertainty regarding the association of genetically-predicted T2D liability with death from cirrhosis, cancer, COPD and other medical or external causes.

**Implications of all the available evidence**

T2D is a strong, causal risk factor for premature death from renal, vascular and infectious diseases in the Mexican population. These strong associations, the high prevalence of T2D, and the prominence of renal, vascular and infectious diseases as causes of premature death in Mexico, demonstrate the importance of T2D primary prevention as well as effective management of T2D and prevention of its associated complications. Such measures would be expected to contribute substantially to reducing premature mortality in this population.

## Introduction

The global burden of diabetes is high and increasing. In 2021, an estimated 0.5 billion adults were living with diabetes, and this number is expected to increase to almost 0.8 billion by 2045.^1^ Type 2 diabetes (T2D) accounts for the vast majority of the current and rising diabetes prevalence, strongly influenced by high levels of excess adiposity.^1^ Previous observational epidemiological studies have found higher risks of diverse diseases associated with T2D, including vascular, renal, chronic liver, respiratory, musculoskeletal and infectious diseases, as well as some cancers.^2^, ^3^, ^4^, ^5^ Given the inherent limitations of observational epidemiology, it is unclear whether these associations reflect a causal effect of T2D, or influences of confounding and other biases, or both. Understanding this better is essential to inform effective disease prevention. By using genetic variants as instrumental variables for modifiable risk factors, such as T2D, Mendelian randomisation can overcome many of the limitations of observational epidemiological analyses, allowing causal inferences to be made.^6^ Findings of previous Mendelian randomisation studies support a causal role for T2D in the aetiology of renal^7^^,^^8^ and vascular^9^, ^10^, ^11^ diseases, but its causal relevance for many other diseases, including infectious,^12^^,^^13^ respiratory,^14^ chronic liver^15^, ^16^, ^17^ and neoplastic^18^ diseases remains uncertain.

In addition to a high prevalence of T2D in Mexico,^1^ previous observational analyses have shown notably strong T2D-associated mortality risks in this population.^5^ Frequently high levels of glycaemia among individuals with T2D, despite treatment with anti-hyperglycaemic agents,^19^ likely in part explain the strength of these relationships. However, lower diabetes-associated mortality risks have been observed in other populations with frequent suboptimal diabetes management.^2^ Mendelian randomisation provides an opportunity to improve our understanding of the factors underlying these previously observed strong mortality associations, including the extent to which they reflect a causal role of T2D, for example, through mechanisms driven by hyperglycaemia, hyperinsulinaemia or related traits such as dyslipidaemia and chronic inflammation, versus the influence of potential confounding factors (e.g., adiposity, lifestyle factors) and uncontrolled biases, such as reverse causality.

Using data from the Mexico City Prospective Study (MCPS) of 150,000 adults, we report the associations of genetically-predicted T2D liability with all-cause and cause-specific mortality, and compare these with associations derived from observational epidemiological analyses.

## Methods

### Study design and participants

Details of the MCPS design, methods and participants have been described previously.^20^ In brief, the baseline survey took place between April 14 1998 and September 28 2004 in Mexico City. Households in two urban districts—Coyoacán and Iztapalapa—were visited and residents aged 35 or older were invited to participate in the study. Of the 112,333 households with eligible residents, at least one individual from 106,059 (94%) households participated. Ethics approval for the study was obtained from the Mexican Ministry of Health, the Mexican National Council for Science and Technology, and the University of Oxford, UK. All participants provided written informed consent.

### Data collection

Participants were interviewed by trained nurses using electronic questionnaires during household visits. Data were collected on sociodemographic characteristics (ethnicity data were not collected), lifestyle factors (including smoking, alcohol drinking and physical activity) and personal medical history, including current medication use. Physical measurements, including blood pressure, height, weight and waist and hip circumferences, were taken using calibrated instruments with standard protocols. A non-fasting venous blood sample was collected into a 10 ml ethylenediaminetetraacetic acid vacutainer and separated into two plasma and one buffy coat aliquots for long-term storage at −150 °C. HbA1c levels were measured in buffy coat samples using validated high-performance liquid chromatography methods^5^ on HA-8180 analysers with calibrators traceable to International Federation of Clinical Chemistry standards.^21^ A high-throughput targeted nuclear magnetic resonance platform^22^ was used to undertake metabolomic profiling in plasma samples.

### Assessment of T2D status

Diabetes was defined as previously diagnosed (self-reported doctor diagnosis of diabetes or diabetes medication use) or undiagnosed (no previously diagnosed diabetes but an HbA1c level of ≥6.5% [48 mmol/mol]^23^) diabetes at recruitment. Participants were considered to have likely type 1 diabetes (T1D) if they reported diagnosis of diabetes before 35 years and were taking insulin at recruitment.

### Genotyping and genetic instrument for T2D

Genotyping of participants was conducted using the Illumina Global Screening array version 2.^24^ Quality control checks included genotype and individual-level missingness, departure from Hardy–Weinberg equilibrium, and Mendel errors.^24^ Proportions of Indigenous American, European, African and East Asian ancestry were estimated using ADMIXTURE software.^24^ After applying quality control criteria,^24^ the resulting genotypes were uploaded to the TOPMED Imputation Server for genome-wide imputation and variants with imputation information scores >0.4 were retained. Genetic principal components were calculated as a measure to enable correction for potential population stratification, and the first 7 genetic principal components were identified as capturing population structure.^25^

The Type 2 Diabetes Global Genomics Initiative (T2D-GGI) meta-analysis of genome-wide association studies (GWAS) reported 1289 independent single nucleotide polymorphisms (SNPs) associated with T2D in a population comprising individuals of diverse ancestries.^26^ Of these, 234 SNPs were ambiguous (n = 203) or were not available (n = 31) in MCPS. The remaining 1055 SNPs were used to construct a T2D genetic risk score (GRS) for each participant as the sum across all SNPs of their effect allele count multiplied by the log odds ratio (OR) for the association of the SNP with T2D in the T2D-GGI multi-ancestry meta-regression (Supplementary Data).^26^ Eight pathway specific T2D GRSs were also constructed, comprising previously-reported non-overlapping subsets of the 1055 T2D-associated SNPs associated with different pathophysiological pathways (beta-cell dysfunction with a positive association with proinsulin, beta-cell dysfunction with a negative association with proinsulin, residual glycaemia, body fat, metabolic syndrome, insulin resistance mediated through obesity, lipodystrophy, and liver and lipid metabolism).^26^ Weights derived from the T2D-GGI multi-ancestry meta-regression were used to construct the main T2D GRS given recommended prioritisation of larger diverse GWAS over smaller ancestry-matched GWAS for GRS construction,^27^ with advantages of the larger effective sample size for risk estimate precision. However, sensitivity analyses used a Hispanic T2D GRS, which was constructed from the same 1055 SNPs, but weighted instead by SNP effects on T2D from Hispanic ancestry groups within the T2D-GGI.^26^

### Follow-up for mortality

Participants are followed for cause-specific mortality through probabilistic linkage (since there was no unique national identification number in Mexico at the time of study recruitment) to the Mexican System for Epidemiologic Death Statistics (Subsistema Epidemiológico y Estadístico de Defunciones or SEED) electronic death registry in Mexico City. Linkage is based on name (including phonetic coding of names), age and sex, and field validation of more than 7000 matched deaths confirmed the reliability of the matching algorithm in more than 95% of deaths. Diseases recorded on death certificates are coded using the International Classification of Diseases, Tenth Revision (ICD-10), and SEED follows World Health Organization conventions^28^ to assign an underlying cause of death. As described previously,^5^^,^^19^ for deaths attributed to diabetes as the underlying cause, study clinicians (blinded to baseline information) review all information recorded in Parts I and II of death certificates, accepting diabetes as the underlying cause only for those deaths occurring during acute diabetic crises. Other deaths attributed to diabetes are re-attributed to a more epidemiologically appropriate underlying cause (e.g., ischaemic heart disease, stroke, peripheral artery disease, chronic kidney disease etc.). This avoids undercounting of deaths from other important causes (e.g., vascular and renal diseases) among people with diabetes.^5^ Participant deaths were tracked until September 2022. ICD-10 codes for the endpoints studied in this paper are shown in Supplementary Table S1.

### Statistical analysis

The main analyses excluded participants aged 75 years or older (both to focus on deaths that could be considered ‘premature’^29^ as well as to minimise any potential biases due to less reliable assessment of underlying cause of death at older ages^30^), those with likely T1D, with missing or extreme exposure or covariate data, or who had an uncertain cause of death. Observational analyses additionally excluded participants with previously diagnosed chronic diseases other than diabetes (ischaemic heart disease, stroke, chronic kidney disease, cirrhosis, cancer, emphysema). Genetic analyses excluded participants with missing genetic data or genetic data failing quality control checks.

In observational analyses, Cox proportional hazards models, with time since entry into the study as the underlying timescale, were used to assess the relevance of previously diagnosed and undiagnosed diabetes (separately and combined) for all-cause and cause-specific (based on underlying cause of death) mortality at 35–74 years. The log hazard ratio from a Cox model provides a useful summary statistic for the average causal log mortality rate ratio (RR) across different time periods of follow-up even if the true log hazard ratios do vary over time (i.e., non-proportionality). These mortality RRs were stratified by age-at-risk (5-year groups) and sex, and adjusted for district (Coyoacán and Iztapalapa), education level (university or high school, middle school, elementary school, other), smoking status (never, former, occasional, <10 cigarettes/day, ≥10 cigarettes/day), alcohol drinking (never, former, current), height (four equal groups), weight (four equal groups), waist circumference (four equal groups) and hip circumference (four equal groups).

Genetic analyses were reported in accordance with Strengthening the Reporting of Observational Studies in Epidemiology using Mendelian Randomisation (STROBE-MR) guidelines.^31^ The study did not have a prospective protocol or published analysis plan, but analyses were planned prior to study initiation. Per allele effects on T2D in MCPS of each of the 1055 SNPs included in the T2D GRS were estimated using logistic regression with adjustment for age, sex and the first 7 genetic principal components, implemented in SUGEN.^32^ These associations were compared with those in the T2D-GGI.^26^ Participant baseline characteristics were assessed across fifths of the T2D GRS distribution. The proportion of T2D genetic liability explained by the GRS was estimated using Nagelkerke's pseudo R^2^.^33^ The ratio of coefficients Mendelian randomisation method^6^ was used to assess the causal relevance of T2D to all-cause and cause-specific mortality at ages 35–74 years, dividing the estimated GRS-outcome associations by estimates of the GRS-T2D association. Cox regression estimated the association between the T2D GRS and mortality (stratified by age-at-risk in 5-year groups and sex, and adjusted for the first 7 genetic principal components), and logistic regression estimated the association between the T2D GRS and T2D (combined diagnosed and undiagnosed T2D) at recruitment (adjusted for age, sex and the first 7 genetic principal components). To aid interpretation, the resulting mortality ln RR estimates and their SEs were multiplied by 1.10 (i.e., ln 3) so that they corresponded to a trebling in the genetically-predicted odds of T2D rather than a ∼2.72-fold increase. (A trebling in *odds* may be thought of as an increase in genetic *risk* of T2D from 10% [odds 1/9] to 25% [odds 1/3], from 25% [odds 1/3] to 50% [odds 1], or from 50% [odds 1] to 75% [odds 3].) Subgroup analyses compared effect sizes across strata of sex, age-at-risk, residential district and proportion of Indigenous American ancestry. For these analyses, the association between the T2D GRS and T2D used in the Mendelian randomisation ratio method was the overall estimate since subgroup-specific estimates would potentially be unreliable due to larger random errors. Additional analyses explored the relevance of pathway specific T2D GRSs to mortality.

Sensitivity analyses included restriction to participants who were unrelated to the third family degree, use of the Hispanic T2D GRS and assessment of the causal relevance of T2D to mortality at ages 75–84 and 35–84 years. The robustness of the Mendelian randomisation results to violations of the instrumental variable assumptions were explored using standard approaches based on summary data using the MendelianRandomization^34^ and MR-PRESSO^35^ R packages. Inverse-variance weighted Mendelian randomisation, the basic summary data approach, assumes all SNPs are valid instrumental variables and that none demonstrates horizontal pleiotropy. MR-Egger provides a robust causal estimate in the presence of directional pleiotropy, assuming it is independent of the association of each SNP with the exposure,^36^ while the weighted median approach provides a robust estimate if at least 50% of the weight in the analyses is derived from SNPs that are valid instrumental variables with no pleiotropic effects.^37^ The Mendelian randomisation Pleiotropy Residual Sum and Outlier (MR-PRESSO)^35^ method identifies and removes SNPs with heterogeneous effects which could suggest pleiotropic effects. These two-sample Mendelian randomisation analyses were based on summary-level data for the 1055 SNPs included in the T2D GRS, using SNP-T2D and SNP-mortality effect estimates and their standard errors in the T2D-GGI multi-ancestry meta-regression^26^ and MCPS, respectively. There was no overlap between these two study populations.

Analyses were conducted using SAS (version 9.4) and R (version 4.3.3).

### Role of the funding source

The funders had no role in study design, data collection, analysis or interpretation, or writing of the report. JAD and JRE had full access to all data in the study and had final responsibility for the decision to submit for publication.

## Results

Of the 159,775 participants recruited, 23,468 were excluded from the main analyses. These comprised 13,648 participants aged 75 years or older at recruitment, a further 262 with likely T1D, 7341 with missing or outlying data, 2025 with uncertain mortality linkage and 192 participants who were recruited more than once (data from the first visit at which a blood sample was collected were used for these participants) (Supplementary Figure S1). Of the remaining 136,287 participants, 14,854 were excluded from the genetic analyses due to missing genetic data or genetic data failing quality control checks while 5751 were excluded from the observational analyses due to a history of prior chronic disease (other than diabetes), leaving 121,433 and 130,536 participants in the genetic and observational analysis populations, respectively.

Among the 121,433 participants in the genetic analysis population, mean (standard deviation [SD]) age was 51 (11) years, 68% (n = 82,249) were women, and, on average, 67% of participants’ genomic ancestry was attributable to Indigenous American populations (Table 1). Mean (SD) body mass index was 29.1 (4.8) kg/m^2^ and 18% (n = 21,371) had T2D at recruitment, including 13% (n = 15,433) with previously diagnosed T2D, who had a mean (SD) HbA1c of 9.1% (2.5%), and 5% (n = 5938) with undiagnosed T2D. Among participants without diabetes, the mean (SD) HbA1c was 5.5% (0.4%). Baseline characteristics of the observational analysis population were very similar (Supplementary Table S2).

**Table 1:** Baseline characteristics of 121,433 participants aged 35–74 years at recruitment.

	Men	Women	Overall
No. of participants	39,184	82,249	121,433
**Age, ancestry and socioeconomic factors**			
Age, years	52 (11)	51 (11)	51 (11)
Indigenous American ancestry, %	67	67	67
Resident of Coyoacán	16,446 (42)	30,462 (37)	46,908 (39)
Resident of Iztapalapa	22,738 (58)	51,787 (63)	74,525 (61)
University or high school educated	9775 (25)	9828 (12)	19,603 (16)
**Lifestyle factors**			
Current smoker	17,828 (45)	17,169 (21)	34,997 (29)
Current alcohol drinker	30,502 (78)	51,866 (63)	82,368 (68)
Physical activity 1+ times/week	11,874 (30)	15,393 (19)	27,267 (22)
**Anthropometric, blood pressure and HbA1c measurements**			
Height, cm	165 (7)	152 (6)	156 (9)
Weight, kg	76.3 (12.6)	68.3 (12.4)	70.8 (13.0)
BMI, kg/m^2^	28.0 (4.1)	29.6 (5.1)	29.1 (4.8)
Waist circumference, cm	96 (10)	93 (12)	94 (11)
Waist-to-hip ratio	0.95 (0.06)	0.88 (0.06)	0.90 (0.07)
Blood pressure, mmHg			
Systolic	128 (15)	126 (17)	127 (16)
Diastolic	84 (10)	82 (10)	83 (10)
HbA1c, %^a^	5.5 (0.4)	5.5 (0.4)	5.5 (0.4)
**Medical history**			
Type 2 diabetes			
Previously diagnosed	5067 (13)	10,366 (13)	15,433 (13)
Undiagnosed	1997 (5)	3941 (5)	5938 (5)
Total	7064 (18)	14,307 (17)	21,371 (18)
Ischaemic heart disease	680 (2)	825 (1)	1505 (1)
Stroke	336 (1)	720 (1)	1056 (1)
Chronic kidney disease	284 (1)	703 (1)	987 (1)
Cirrhosis	105 (<0.5)	55 (<0.5)	160 (<0.5)
Cancer	195 (<0.5)	1164 (1)	1359 (1)
Emphysema	129 (<0.5)	128 (<0.5)	257 (<0.5)
**Long-term medication use**			
Any diabetes medication	3879 (10)	8350 (10)	12,229 (10)
Any anti-hypertensive medication	4024 (10)	13,147 (16)	17,171 (14)
Any lipid-lowering medication	244 (1)	415 (1)	659 (1)
Any anti-thrombotic medication	1030 (3)	2251 (3)	3281 (3)

Mean (SD) or n (%).

BMI, body mass index.

a Among participants without diabetes.

**Fig. 1:**
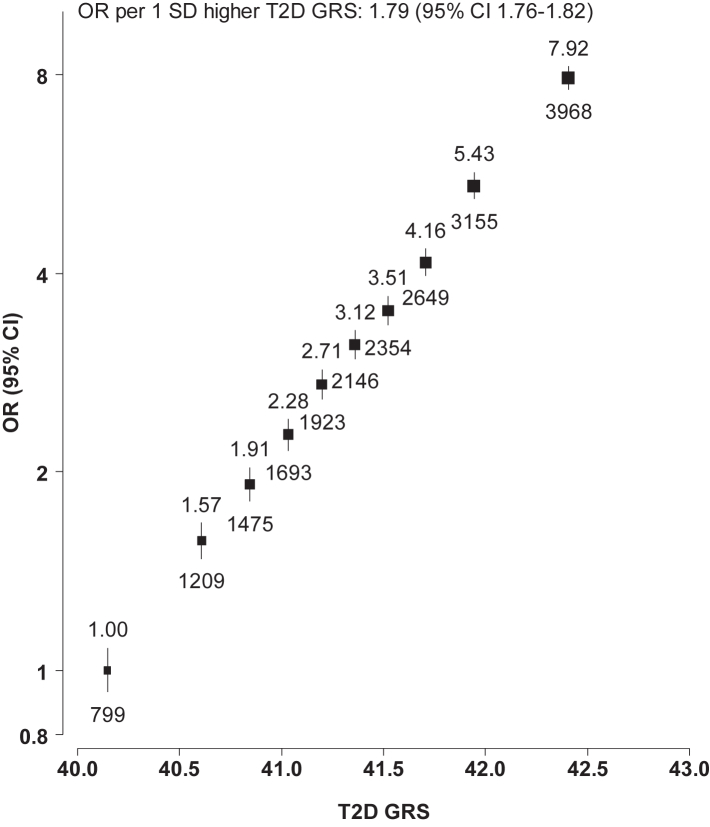
**Association of type 2 diabetes GRS with type 2 diabetes at recruitment**. Type 2 diabetes (T2D) at recruitment refers to previously diagnosed or undiagnosed T2D. The T2D genetic risk score (GRS) represents the sum across all SNPs of their effect allele count multiplied by the log odds ratio (OR) for the association of the SNP with T2D in the T2D Global Genomics Initiative multi-ancestry meta-regression. T2D GRS SD = 0.64 units. T2D ORs are adjusted for age, sex and the first 7 genetic principal components. For each of the 10 equally-sized categories, the numbers above the squares are the ORs and the numbers below the squares are the number of participants with T2D in that group. The area of each square is proportional to the variance of the log odds for that group (including for the reference group with OR = 1.00). Error bars represent 95% confidence intervals (CI).

Associations with T2D in the MCPS population of the 1055 individual SNPs included in the T2D GRS correlated moderately well with associations in the T2D-GGI multi-ancestry meta-regression (r = 0.47; Supplementary Figure S2). The GRS was a strong instrument, explaining 6.3% of T2D genetic liability. Each 1 SD increment in the GRS was associated with a T2D OR of 1.79 (95% confidence interval [CI] 1.76–1.82) (Fig. 1). This association was slightly stronger among women than men, at younger ages, and among individuals with a lower proportion of Indigenous American ancestry (Supplementary Figure S3). Mean HbA1c levels at recruitment increased across fifths of the T2D GRS, but the GRS was not associated with other traits, including potential confounders of the association of T2D with mortality (Supplementary Table S3). The Hispanic T2D GRS was only slightly less strongly associated with T2D (OR 1.76 [95% CI 1.67–1.87]) than the multi-ancestry GRS (Supplementary Figure S4).

During a median (interquartile range) of 20.2 (19.4–21.4) years’ follow-up, 12,293 participants died at ages 35–74 among the 121,433 participants included in the main genetic analyses. This included 3226 deaths from vascular disease (including 2287 cardiac and 695 stroke deaths), 2437 from infectious disease, 1696 from renal disease, 2016 from cancer, 895 from cirrhosis, 287 from chronic obstructive pulmonary disease, and 509 from acute diabetic crises. There were 12,573 deaths at 35–74 years among the 130,536 participants included in observational analyses.

**Fig. 2:**
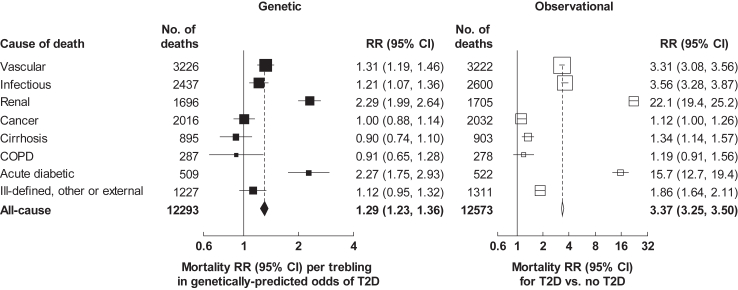
**Genetic and observational associations of type 2 diabetes with cause-specific mortality at ages 35–74 years**. Genetic mortality rate ratios (RRs) are per trebling in the genetically-predicted odds of type 2 diabetes (T2D) and are stratified by age-at-risk and sex and adjusted for the first 7 genetic principal components. T2D in observational associations refers to previously diagnosed or undiagnosed T2D. Observational mortality RRs are stratified by age-at-risk and sex and adjusted for district, educational level, smoking status, alcohol drinking, height, weight, waist circumference and hip circumference. The size of each square is inversely proportional to the variance of the log RR. Horizontal lines represent 95% confidence intervals (CI). COPD, chronic obstructive pulmonary disease.

Fig. 2 shows RRs for death from any cause and from several major underlying causes associated with a trebling in the genetically-predicted odds of T2D (RRs associated with 1-unit higher log-odds of genetic liability to T2D are shown in Supplementary Figure S5). The RR for death from any cause associated with a trebling in genetically-predicted odds of T2D was 1.29 (95% CI 1.23–1.36). There was a particularly strong association with death from renal disease and from acute diabetic crises, with mortality RRs of 2.29 (95% CI 1.99–2.64) and 2.27 (1.75–2.93), respectively. This was consistent with strong relationships of T2D with death from renal disease (RR 22.1 [95% CI 19.4–25.2]) and acute diabetic crises (15.7 [12.7–19.4]) in observational analyses (reflecting the combined associations of previously diagnosed and undiagnosed T2D; Supplementary Figure S6). Trebling in the genetically-predicted odds of T2D was associated with a 31% increase in the risk of death from vascular disease overall (RR 1.31 [95% CI 1.19–1.46]), with similar mortality RRs for vascular disease subtypes (Supplementary Figure S7). There was a similar risk of death from infection (RR 1.21 [95% CI 1.07–1.36]) (Fig. 2), reflecting strong associations of genetically-predicted T2D liability with death from septicaemia (1.84 [1.20–2.82]) and from urinary tract infection (1.61 [1.07–2.42]), and more modest associations with death from other specific infections (Supplementary Figure S8).

There was no apparent association of genetically-predicted T2D liability with death from cancer overall (RR 1.00 [95% CI 0.88–1.14]) (Fig. 2), reflecting varied associations with site-specific cancers (Supplementary Figure S9), although there was a possible modest association of T2D with cancer mortality in observational analyses (1.12 [1.00, 1.26]). Genetically-predicted T2D liability showed no clear association with death from chronic obstructive pulmonary disease (RR 0.91 [95% CI 0.65–1.28]), consistent with observational findings. T2D did not appear to be associated with death from cirrhosis in genetic analyses (RR 0.90 [95% CI 0.74–1.10]), in contrast with a modest positive observational association (1.34 [1.14–1.57]). Similarly, there was no clear association of genetically-predicted T2D liability with death from ill-defined, other medical or external causes, despite apparent positive associations in observational analyses (Supplementary Figure S10).

**Fig. 3:**
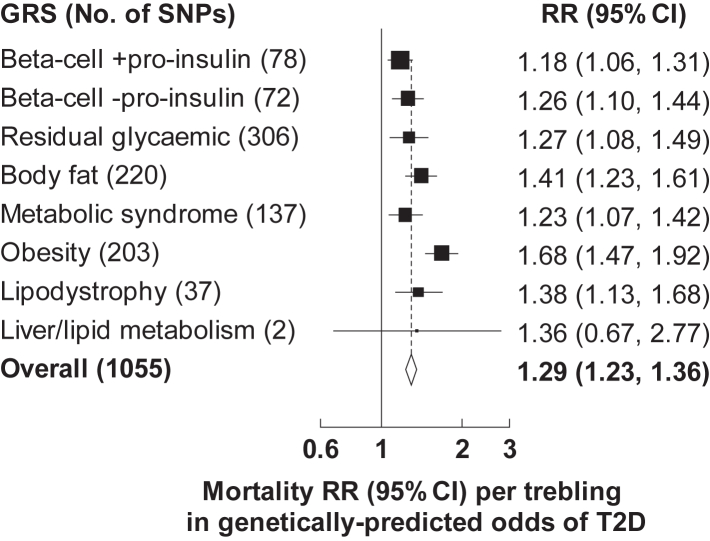
**Relevance of pathway specific type 2 diabetes GRSs to all-cause mortality at ages 35–74 years**. Mortality rate ratios (RRs) are per trebling in the genetically-predicted odds of type 2 diabetes (T2D) and are stratified by age-at-risk and sex and adjusted for the first 7 genetic principal components. The size of each square is inversely proportional to the variance of the log RR. Horizontal lines represent 95% confidence intervals (CI). GRS, genetic risk score; SNP, single nucleotide polymorphism.

When pathway-specific T2D GRSs were examined, all were associated with risk of death from any cause (the finding for the liver/lipid metabolism pathway GRS should be interpreted with caution since it included only 2 SNPs) (Fig. 3). The obesity pathway T2D GRS was more strongly associated with overall mortality (RR 1.68 [95% CI 1.47–1.92] per trebling in the genetically-predicted odds of T2D) than the other pathway specific GRSs, largely driven by differences seen for mortality from vascular and infectious diseases (Supplementary Figure S11).

For renal disease, the premature death RR associated with genetically-predicted T2D liability was larger at younger ages, but there were no clear differences in age-specific death RRs for other causes of premature death (Supplementary Figure S12). Genetically-predicted T2D liability was slightly less strongly associated with cause-specific mortality—most notably renal and vascular mortality—at ages 75–84 than at younger ages (Supplementary Figure S13). Among participants aged 35–84, trebling in genetically-predicted odds of T2D was associated with a RR for death from any cause of 1.24 (95% CI 1.18–1.29), and 1.24 (1.14–1.34), 1.17 (1.07–1.29), 2.10 (1.85–2.37) and 2.13 (1.72–2.63) for death from vascular, infectious, renal and acute diabetic causes, respectively (Supplementary Figure S14). Mortality associations of genetically-predicted T2D liability were similar in men and women (Supplementary Figure S15), across residential districts (Supplementary Figure S16), and among participants who were unrelated up to the third family degree (Supplementary Figure S17). Moreover, there were no clear differences according to the proportion of Indigenous American ancestry (Supplementary Figure S18) or when analyses were repeated using the Hispanic T2D GRS (Supplementary Figure S19).

MR-PRESSO, weighted median and MR-Egger methods showed mortality associations of genetically-predicted T2D liability comparable with those in the main analyses (Supplementary Table S4), supporting the robustness of the presented findings against violations of instrumental variable assumptions. Although MR-Egger analyses provided some evidence for a non-zero intercept for all-cause mortality (p = 0.001), the intercept (1.004 [95% CI 1.002–1.006]) and potential for directional pleiotropy were small.

## Discussion

In this large study in a Mexican population, genetically-predicted T2D liability was associated with premature vascular, renal and infectious disease mortality and was not associated with the major potential confounders of these relationships, supporting a causal role for T2D in death from these causes. The association with renal deaths was strongest, for which a trebling in the genetically-predicted odds of T2D was associated with more than a doubling in mortality risk. Associations with vascular and infectious disease mortality were more modest but still strong. Beyond the expected causal relationship between T2D and death from acute diabetic crises, there was no apparent association of genetically-predicted T2D liability with death from other major underlying causes, including cancer and cirrhosis.

A causal role for T2D in vascular and renal diseases is supported by findings of previous Mendelian randomisation studies,^7^, ^8^, ^9^, ^10^, ^11^ and may variably reflect hyperglycaemia, inflammation, endothelial dysfunction, oxidative stress and lipid related mechanisms.^8^^,^^38^ However, previous studies have tended to show weaker associations than those observed in the present study. For example, in one multi-ethnic study, a doubling in the genetically-predicted odds of T2D was associated with ORs for chronic kidney disease of 1.08 and 1.07 in European and East Asian ancestry populations, respectively.^8^ This is substantially weaker than the association with renal mortality in MCPS (RR 1.69 after applying the same transformation). Similarly, in a large two-sample European ancestry Mendelian randomisation study, each 1-unit higher log-odds of genetically-predicted risk of T2D was associated with 13% higher odds of ischaemic heart disease,^11^ notably smaller than the 27% increase in risk of cardiac mortality in MCPS. The stronger associations in the MCPS population might reflect the focus on mortality outcomes (previous studies have tended to investigate combined fatal and non-fatal disease outcomes),^7^, ^8^, ^9^, ^10^, ^11^ or differential effects of T2D by ancestry, including disparities in T2D pathophysiology and epidemiology. Pleiotropic effects of T2D-associated genetic variants might also contribute. Sensitivity analyses provided no evidence of strong horizontal pleiotropic effects, which would also be expected to be limited by inclusion in the T2D GRS of SNPs acting through various pathophysiologic pathways. However, the higher causal estimates in MCPS could reflect vertical pleiotropic effects, for example, due to frequently suboptimal T2D management in Mexico.^39^

There is uncertainty regarding the causal relevance of T2D for infectious diseases. In a two-sample Mendelian randomisation study based on summary statistics from European ancestry GWAS, no association was observed between genetically-predicted T2D liability and any of the five specific infectious diseases studied.^12^ However, findings from studies of COVID-19 suggest T2D may specifically play a role in determining prognosis, rather than initial development, of infection. For example, a European ancestry two-sample Mendelian randomisation analysis found no convincing evidence for a causal effect of T2D on SARS-CoV-2 infection (OR 1.02 [95% CI 1.00–1.03] per 1-unit higher log-odds of genetically-predicted risk of T2D), but positive associations with COVID-19 hospitalisation (1.08 [1.04–1.22]) and critical COVID-19 (i.e., requiring respiratory support or causing death) (1.09 [1.03–1.16]).^13^ Hyperglycaemia and immune dysfunction related pathways could contribute to these causal associations of T2D with infectious disease outcomes.^13^^,^^40^ It is unclear whether the association between genetically-predicted T2D liability and death from infection in the present study reflects the influence of T2D on onset or progression of infectious diseases, but the strong association with death from sepsis could support the latter assertion. Irrespective of the specific role of T2D, the findings highlight the importance of efforts to prevent infectious diseases in this population group.

Although observational analyses suggested a modest 34% higher risk of death from cirrhosis associated with T2D in the MCPS population, there was no apparent association in genetic analyses. This could reflect limited statistical power given the relatively small number of deaths from cirrhosis and findings of a previous, much larger Mendelian randomisation study (including 5904 cirrhosis cases and 706,200 controls) which showed a clear association with cirrhosis (OR 1.12 per 1-unit higher log-odds of genetically-predicted risk of T2D).^15^ However, differences between studies in the apparent causal relevance of T2D could also reflect varying aetiologies underlying cirrhosis diagnoses.

Mendelian randomisation provides understanding of causality, but often does not afford detailed clarification of underlying mechanisms. However, the varied strengths of association of pathway specific T2D GRSs with mortality in the present study, including apparently stronger associations of the insulin resistance mediated obesity pathway GRS with overall mortality and with death from vascular and infectious diseases, may offer some initial pathophysiological insights. Previous studies have observed similar strong associations of obesity-related pathways with vascular complications of T2D,^26^^,^^41^ but this has not previously been reported for infectious diseases. These findings suggest targeting obesity-related pathways associated with T2D may be particularly effective for prevention of premature vascular and infectious disease deaths in T2D. Moreover, such analytical approaches could ultimately inform risk stratification, personalised medicine and drug development efforts; collectively, these findings warrant further investigation. However, even without a clear understanding of underlying mechanisms, the current study's findings can help to inform policies for reducing T2D-associated premature mortality in Mexico. For example, through primary prevention, targeting, at individual and population levels, the drivers of high T2D prevalence in Mexico, including excess adiposity,^42^ as well as through effective management of T2D and prevention of its associated complications.

In contrast with most previous studies, we employed a one-sample Mendelian randomisation approach based on individual participant data. This enabled assessment of the association of the genetic instrument with T2D and with mortality risks in the same population, as well as permitting valuable subgroup analyses, showing, for example, comparable associations of genetically-predicted T2D liability with cause-specific mortality among men and women. Moreover, we were able to explore observational and genetic associations of T2D in the same population. Well-described challenges of investigating binary exposures in Mendelian randomisation analyses^43^ prevented direct comparison of the strength of genetic and observational associations in the present study. However, for the first time, the presented analyses provide valuable genetic evidence of the contribution of strong causal effects of T2D to the higher T2D-associated renal, vascular and infectious disease mortality risks observed in the Mexican population,^5^^,^^19^ when compared with other populations globally.^2^^,^^4^ Furthermore, this is the only study to-date to examine the genetic relevance of T2D for the full range of cause-specific mortality in a single population, enabling direct comparison of the strength of causal relationships of T2D with different diseases. Although the focus on a Mexican population could limit wider applicability of our findings, comparable associations of multi-ancestry and Hispanic ancestry GRSs support generalisability of the causality of relationships to other populations. Construction of the T2D GRS based on findings from a large multi-ancestry GWAS facilitated precise estimates of mortality risks, but there is also a clear need for large-scale ancestry-specific GWAS which are currently lacking for Hispanic populations. The one-sample Mendelian randomisation findings were largely supported by summary-level data Mendelian randomisation, suggesting the estimated causal effects of T2D on cause-specific mortality were robust to directional pleiotropy and heterogeneous SNP-specific causal effects, although modest directional pleiotropy may have contributed to the association with all-cause mortality. However, our study has certain limitations. As described, the outcomes studied were restricted to mortality. Moreover, there was limited statistical power to assess reliably the genetic associations of T2D with some causes of death (e.g., cancer subtypes). We were unable to access medical records for validation of previously diagnosed diabetes. However, high average HbA1c levels support the validity of these self-reported diagnoses, and inclusion of undiagnosed diabetes, identified through HbA1c measurement, would be expected to minimise diabetes status misclassification. The study included more women than men, reflecting recruitment through household visits mostly during standard working hours when women were more likely to be at home. However, sufficiently large numbers of both men and women were included to enable reliable and generalisable sex-specific estimates of the associations of genetically-predicted T2D liability with major cause-specific mortality. Finally, although extensive sensitivity analyses and the strong T2D genetic instrument demonstrate the robustness of our findings, including against violations of the instrumental variable assumptions, some residual horizontal pleiotropy cannot be ruled out.

In summary, our findings provide evidence in support of strong causal effects of T2D on death from vascular, renal and infectious diseases in Mexican adults. The high prevalence of T2D in Mexico, coupled with notably strong causal relationships with these major causes of premature death, demonstrate the importance of preventing and effectively managing T2D, including through prevention and treatment of vascular, renal and infectious complications. This would be expected to have substantial benefits for rates of premature mortality in Mexico.

## Contributors

*Establishing the cohort:* PKM, JAD and RTC. *Obtaining funding and study enhancement*: PKM, RC, RP, JRE, JAD and RTC. *Data acquisition, analysis, or interpretation of data*: All authors. *Laboratory support*: MH. *Drafting first version of manuscript*: FB. *Critical revision of the report for important intellectual content:* All authors. All authors have seen and approved the final version and agreed to its publication. JRE and JAD had full access to all the data in the study, verified the underlying data, and take responsibility for the integrity of the data and the accuracy of the analysis.

## Data sharing statement

Data from the Mexico City Prospective Study are available to bona fide academic researchers. For more details, the study's Data and Sample Sharing policy may be downloaded (in English or Spanish) from https://www.ctsu.ox.ac.uk/research/mcps. Available study data can be examined in detail through the study's Data Showcase, available at https://datashare.ndph.ox.ac.uk/mexico/. MCPS ancestry-specific allele frequencies are available in a public browser (https://rgc-mcps.regeneron.com/).

## Declaration of interests

JRE and RC report grants to the University of Oxford from AstraZeneca and Regeneron Pharmaceuticals. RC reports having a patent for a statin-related myopathy genetic test licensed to the University of Oxford from Boston Heart Diagnostics (RC has waived any personal reward with any share in royalty and other payments waived in favour of the Nuffield Department of Population Health, University of Oxford) and being deputy chair of not-for-profit clinical trial company PROTAS, Chief Executive of UK Biobank, and Chair of the steering committee of the ORION-4 clinical trial of inclisiran. WGH reports a personal fellowship from Kidney Research UK, grants to the University of Oxford from Boehringer Ingelheim and Eli Lilly, and being co-chair of the European Society of Cardiology Guideline Task Force. All other authors declare no competing interests.
