# Supplementary material for: Type 2 diabetes and cause-specific mortality in Mexico City: a Mendelian randomisation analysis
Source: Lancet Reg Health Am. 2025 Apr 6;45:101082. doi: 10.1016/j.lana.2025.101082 (PMC12001093; doi:10.1016/j.lana.2025.101082)
Supplement: Online Supplement [file mmc1.pdf]

Type 2 diabetes (T2D) and cause-specific mortality in Mexico City: a Mendelian randomisation analysis

Supplementary Material, Table of Contents

Supplementary tables	Page
1. Number of deaths at ages 35-74 years by underlying cause (ICD-10 code) among 121,433 participants aged 35-74 years at recruitment	2
2. Baseline characteristics of 130,536 participants (observational analysis population) aged 35-74 years at recruitment	5
3. Baseline characteristics of 121,433 participants aged 35-74 years at recruitment by fifths of T2D GRS	6
4. Associations of T2D with risk of mortality using robust Mendelian randomisation methods based on summary-level data	7

Supplementary figures

1. Participant exclusions to derive genetic and observational analysis populations	8
2. Genetic variant effect size estimates for T2D in Mexico City Prospective Study and in T2D Global Genomics Initiative multi-ancestry GWAS	9
3. Association of T2D GRS with T2D at recruitment by sex, age and Indigenous American ancestry proportion	10
4. Association of Hispanic T2D GRS with T2D at recruitment	11
5. Cause-specific mortality rate ratios per 1-unit higher log-odds of genetically-predicted T2D at ages 35-74 years	12
6. Observational associations of previously diagnosed and undiagnosed T2D with cause-specific mortality at ages 35-74 years	13
Genetic and observational associations of T2D with:	
7. Vascular mortality at ages 35-74 years	14
8. Infectious mortality at ages 35-74 years	15
9. Cancer mortality at ages 35-74 years	16
10. Ill-defined, other medical or external mortality at ages 35-74 years	17
11. Relevance of pathway specific T2D GRS to cause-specific mortality at ages 35-74 years	18
12. Genetic associations of T2D with cause-specific mortality, by age-at-risk	19
13. Genetic and observational associations of T2D with cause-specific mortality at ages 75-84 years	20
14. Genetic and observational associations of T2D with cause-specific mortality at ages 35-84 years	21
Genetic associations of T2D with cause-specific mortality at ages 35-74 years:	
15. By sex	22
16. By district	23
17. By relatedness	24
18. By Indigenous American ancestry proportion	25
19. Associations of multi-ancestry and Hispanic T2D GRSs with cause-specific mortality at ages 35-74 years	26

Supplementary Table 1: Number of deaths at ages 35-74 years by underlying cause (ICD-10 code) among 121,433 participants aged 35-74 years at recruitment

Underlying cause of death	ICD-10 codes (number of such deaths recorded by 30 Sept 2022)
**Vascular**	
Cardiac	I011 (1), I018 (1), I050 (2), I051 (1), I059 (18), I069 (2), I070 (1), I071 (1), I079 (1), I080 (3), I081 (1), I091 (1), I099 (15), I110 (89), I119 (9), I200 (3), I209 (3), I210 (20), I211 (11), I213 (1), I219 (1650), I220 (2), I221 (1), I229 (1), I249 (30), I251 (44), I252 (1), I258 (9), I259 (88), I270 (3), I272 (3), I279 (7), I319 (1), I330 (7), I340 (3), I350 (10), I351 (1), I358 (2), I38X (8), I420 (10), I421 (1), I426 (1), I429 (1), I442 (9), I443 (2), I460 (1), I469 (7), I471 (2), I472 (3), I489 (3), I48X (6), I490 (6), I499 (6), I500 (49), I501 (11), I509 (65), I515 (2), I518 (3), I519 (9), Q210 (1), Q213 (1), Q238 (1), R570 (32)
Cerebrovascular	F019 (2), I600 (2), I602 (1), I608 (1), I609 (73), I61 (1), I610 (1), I612 (1), I613 (1), I614 (1), I615 (2), I618 (1), I619 (208), I620 (6), I629 (4), I633 (4), I634 (16), I635 (3), I638 (1), I639 (48), I64X (88), I669 (4), I671 (5), I672 (1), I673 (1), I674 (2), I678 (77), I679 (96), I691 (1), I693 (8), I694 (6), I698 (29)
Other vascular	E115 (28), E145 (25), I260 (2), I269 (71), I710 (4), I712 (1), I713 (4), I718 (3), I729 (2), I731 (1), I739 (3), I740 (1), I741 (1), I743 (1), I771 (14), I776 (1), I779 (1), I802 (5), I803 (1), I822 (1), I829 (1), I839 (1), I872 (4), I879 (1), I890 (1), I99X (3), K550 (56), K551 (1), K552 (1), K559 (4), K761 (1)
**Infectious**	
Urinary tract	N10X (1), N151 (10), N300 (1), N309 (1), N390 (185), N498 (5)
Skin, bone and connective tissue	L021 (3), L022 (4), L023 (2), L024 (2), L031 (5), L038 (1), L039 (3), L089 (36), L899 (7), L89X (3), L984 (6), M009 (1), M600 (2), M725 (1), M726 (17), M798 (21), M869 (3)
Septicaemia	A415 (1), A419 (180), A483 (1), R572 (4)
Gastrointestinal	A047 (3), A060 (1), A090 (17), A099 (31), A09X (10), A183 (1), B462 (1), K052 (1), K352 (4), K353 (4), K358 (4), K359 (2), K37X (1), K572 (2), K578 (5), K579 (12), K610 (3), K612 (1), K650 (26), K658 (1), K659 (49)
Respiratory	A162 (7), A165 (1), A169 (2), B206 (2), B440 (1), B441 (1), B909 (1), J069 (1), J09 (2), J09X (1), J100 (2), J110 (1), J111 (1), J129 (4), J151 (3), J157 (1), J159 (33), J180 (49), J181 (25), J182 (2), J189 (551), J209 (4), J22X (15), J348 (1), J391 (1), J850 (2), J852 (2), J869 (7), U071 (484), U072 (282), U099 (1)
Other	A170 (1), A181 (2), A182 (1), A199 (3), A498 (1), A810 (2), A86X (2), B200 (1), B201 (1), B207 (4), B208 (7), B210 (2), B212 (1), B218 (1), B227 (2), B238 (4), B24X (4), B259 (1), B373 (1), B451 (1), B465 (2), B690 (2), B948 (2), B99X (1), G009 (3), G039 (3), G042 (1), G049 (7), G060 (1), H440 (1), H669 (1), K223 (2), K228 (1), K251 (3), K254 (8), K255 (10), K256 (4), K259 (4), K264 (5), K265 (3), K269 (2), K274 (2), K275 (1), K290 (6), K291 (1), K292 (1), K295 (5), K297 (1), K318 (4), K920 (24), K921 (4), K922 (102), N719 (1), N739 (1)
**Renal**	E102 (8), E112 (790), E142 (315), I120 (102), I130 (3), I131 (2), I132 (23), N002 (1), N009 (8), N039 (25), N049 (1), N059 (11), N12X (12), N142 (1), N179 (70), N180 (11), N185 (31), N189 (208), N19X (61), N200 (5), N201 (1), N281 (1), N289 (5), Y841 (1)

Underlying cause of death	ICD-10 codes (number of such deaths recorded by 30 Sept 2022)
**Cancer**	
Lung	C33X (1), C349 (131), D380 (2), D381 (5), D383 (2)
Liver, gallbladder or bile duct	C220 (47), C221 (21), C229 (99), C23X (23), C240 (12), C241 (9), C248 (3), C249 (14), D376 (6)
Urinary tract	C64X (84), C679 (22), C680 (2), C689 (1), D410 (3)
Colorectal	C170 (10), C179 (1), C182 (3), C184 (1), C187 (2), C189 (106), C19X (4), C20X (20), C211 (1), D372 (1), D374 (6)
Oesophageal/stomach	C159 (15), C160 (3), C169 (191), D371 (6)
Cervix	C530 (1), C539 (111)
Female breast	C509 (226), D486 (1)
Haematological	C817 (1), C819 (9), C822 (1), C829 (1), C833 (13), C838 (1), C839 (3), C844 (1), C845 (3), C851 (1), C857 (2), C859 (41), C900 (39), C901 (1), C902 (1), C910 (20), C911 (2), C920 (26), C921 (7), C927 (2), C929 (3), C950 (2), C959 (2)
Prostate	C61X (67)
Endometrial	C541 (22), C549 (2), C55X (11)
Pancreas	C250 (20), C252 (1), C259 (82)
Ovary	C519 (3), C52X (1), C56X (98), D391 (4), D397 (1)
Upper aerodigestive	C029 (11), C07X (2), C089 (1), C109 (2), C139 (1), C140 (5), C142 (1), C329 (12)
Other defined site	C319 (1), C37X (1), C382 (1), C383 (1), C384 (1), C412 (2), C419 (6), C435 (1), C437 (1), C438 (1), C439 (10), C444 (1), C445 (1), C447 (1), C449 (7), C451 (2), C457 (1), C459 (4), C469 (1), C479 (1), C480 (8), C482 (4), C492 (4), C495 (1), C499 (13), C509 (1), C609 (2), C629 (1), C694 (1), C709 (2), C710 (12), C711 (1), C718 (2), C719 (38), C720 (1), C729 (1), C73X (26), C741 (1), C749 (1), C97X (1), D377 (2), D429 (1), D430 (14), D432 (1), D449 (1), D487 (8)
Ill-defined	C260 (3), C269 (2), C760 (6), C761 (1), C762 (3), C763 (2), C764 (1), C765 (2), C780 (7), C786 (5), C787 (7), C788 (2), C793 (2), C794 (3), C796 (1), C798 (3), C800 (26), C809 (17), C80X (2), D489 (3)
**Cirrhosis**	B169 (3), B171 (28), B182 (11), B189 (1), B190 (2), B199 (2), I850 (16), I859 (6), K701 (13), K702 (1), K703 (158), K704 (23), K709 (16), K711 (2), K716 (1), K720 (9), K721 (56), K729 (199), K739 (2), K742 (1), K743 (2), K745 (1), K746 (278), K750 (14), K754 (4), K759 (2), K764 (1), K766 (11), K767 (14), K768 (1), K769 (16), Q447 (1)
**COPD**	J42X (11), J439 (27), J440 (88), J441 (2), J448 (6), J449 (153)
**Acute diabetic**	E100 (3), E110 (149), E111 (175), E140 (96), E141 (79), E162 (7)
**Ill-defined, other or external**	
Other medical	D033 (1), D136 (1), D27X (1), D320 (1), D329 (9), D352 (1), D420 (1), D467 (1), D469 (6), D471 (1), D474 (1), D479 (1), D619 (4), D649 (5), D682 (1), D693 (2), D694 (1), D696 (2), D699 (3), D70X (2), D733 (1), D762 (1), E035 (1), E039 (11), E049 (1), E055 (1), E059 (3), E065 (1), E116 (3), E119 (8), E129 (1), E146 (3), E149 (5), E230 (1), E249 (2), E279 (1), E43X (1), E440 (1), E660 (1), E725 (1), E848 (1), E875 (2), E876 (1), E878 (1), E889 (2), F03X (5), F09X (1), F102 (4), F182 (1), F209 (2), G10X (6), G121 (1), G122 (17), G20X (5), G231 (1), G300 (1), G309 (1), G310 (1), G35X (1), G403 (1), G409 (10), G419 (1), G439 (1), G589 (1), G610 (4), G709 (1), G710 (2), G809 (1), G822 (1), G919 (4), G931 (9), G934 (5), G935 (1), G936 (2), G938 (1), G958 (1), I10X (3), J459 (9), J46X (2), J47X (2), J60X (1), J64X (4), J677 (1), J679 (2), J680 (1), J690 (2), J80X (4), J81X (1), J841 (67), J848 (1), J849 (10), J90X (5), J939 (1), J942 (2), J960 (5), J961 (1), J969 (3), J980 (1), J984 (15), J985 (4), J988 (7), J989 (1), K088 (1), K102 (1), K137 (1), K389 (1), K403 (1), K404 (1), K409 (1), K419 (1), K420 (4),

Underlying cause of death	ICD-10 codes (number of such deaths recorded by 30 Sept 2022)
	K429 (2), K430 (1), K439 (2), K440 (1), K460 (3), K461 (1), K469 (2), K513 (1), K519 (1), K529 (6), K560 (2), K562 (1), K566 (36), K593 (5), K628 (1), K630 (1), K631 (22), K632 (4), K635 (1), K638 (1), K639 (5), K661 (1), K800 (4), K801 (9), K802 (4), K803 (3), K804 (1), K805 (2), K810 (10), K811 (3), K819 (4), K822 (2), K829 (3), K830 (17), K831 (3), K839 (1), K851 (1), K852 (4), K858 (7), K859 (30), K85X (24), K861 (3), K868 (2), K918 (1), K931 (1), L100 (1), L109 (1), L511 (2), L512 (1), L921 (1), L958 (1), L988 (1), M050 (1), M068 (1), M069 (16), M100 (1), M109 (1), M139 (1), M311 (2), M313 (1), M319 (1), M321 (3), M329 (1), M340 (1), M349 (1), M469 (1), M623 (5), M799 (1), N40X (8), N948 (1), O720 (1), Q311 (1)
External	S729 (2), T07X (1), T874 (1), V011 (1), V029 (1), V041 (1), V051 (1), V093 (3), V099 (61), V182 (1), V209 (1), V299 (1), V439 (1), V489 (1), V496 (1), V499 (12), V580 (1), V719 (1), V785 (1), V878 (6), V892 (10), V899 (3), W018 (1), W100 (13), W104 (1), W105 (1), W108 (2), W126 (1), W130 (9), W134 (2), W139 (2), W170 (4), W172 (1), W174 (1), W176 (1), W178 (2), W179 (1), W18 (1), W180 (4), W181 (1), W184 (2), W188 (1), W190 (7), W194 (1), W195 (1), W199 (3), W206 (1), W250 (1), W314 (1), W340 (1), W370 (1), W744 (2), W748 (3), W769 (1), W780 (1), W789 (1), W799 (1), W849 (1), W878 (1), X09 (1), X090 (3), X094 (1), X099 (1), X219 (1), X314 (1), X360 (1), X459 (1), X470 (1), X590 (2), X594 (1), X598 (1), X599 (24), X650 (1), X680 (1), X700 (5), X740 (3), X780 (3), X800 (1), X910 (1), X914 (2), X950 (3), X954 (17), X955 (1), X959 (1), X990 (6), X994 (5), X999 (2), Y044 (1), Y048 (1), Y084 (1), Y099 (1), Y159 (1), Y200 (1), Y240 (1), Y244 (3), Y245 (1), Y248 (1), Y249 (2), Y260 (2), Y280 (1), Y330 (1), Y334 (1), Y338 (1), Y340 (5), Y344 (4), Y346 (1), Y348 (2), Y349 (13), Y405 (1), Y579 (3), Y838 (1), Y839 (4), Y846 (1)
Ill-defined	E86X (3), E870 (1), E872 (9), E874 (1), R040 (1), R100 (3), R11X (1), R190 (1), R568 (1), R571 (16), R579 (2), R58X (3), R688 (19), R69X (1), R99X (172)

Supplementary Table 2: Baseline characteristics of 130,536 participants (observational analysis population) aged 35-74 years at recruitment

	Men	Women	Overall
No. of participants	42,284	88,252	130,536
**Age, ancestry and socioeconomic factors**			
Age, years	51 (11)	50 (10)	51 (11)
Indigenous American ancestry, %	67	67	67
Resident of Coyoacán	18,215 (43)	33,356 (38)	51,571 (40)
Resident of Iztapalapa	24,069 (57)	54,896 (62)	78,965 (60)
University or college educated	10,561 (25)	10,608 (12)	21,169 (16)
**Lifestyle factors**			
Current smoker	19,507 (46)	18,455 (21)	37,962 (29)
Current alcohol drinker	33,232 (79)	55,964 (63)	89,196 (68)
Physical activity 1+ times/week	12,708 (30)	16,382 (19)	29,090 (22)
**Anthropometric, blood pressure and HbA1c measurements**			
Height, cm	165 (7)	152 (6)	156 (9)
Weight, kg	76.2 (12.6)	68.2 (12.4)	70.8 (13.0)
BMI, kg/m^2	28.0 (4.1)	29.6 (5.1)	29.1 (4.2)
Waist circumference, cm	96 (10)	93 (12)	94 (11)
Waist-to-hip ratio	0.95 (0.06)	0.88 (0.06)	0.90 (0.07)
Blood pressure, mmHg			
Systolic	128 (15)	126 (16)	126 (16)
Diastolic	84 (10)	82 (10)	83 (10)
HbA1c, %*	5.5 (0.4)	5.5 (0.4)	5.5 (0.4)
**Medical history**			
Type 2 diabetes			
Previously diagnosed	5228 (12)	10,769 (12)	15,997 (12)
Undiagnosed	2134 (5)	4211 (5)	6345 (5)
Total	7362 (17)	14,980 (17)	22,342 (17)
**Long-term medication use**			
Any diabetes medication	4014 (10)	8687 (10)	12,701 (10)
Any anti-hypertensive medication	3740 (9)	13,339 (15)	17,079 (13)
Any lipid-lowering medication	227 (1)	421 (<0.5)	648 (<0.5)
Any anti-thrombotic medication	837 (2)	2155 (2)	2992 (2)

Mean (SD) or n (%)
*Among participants without diabetes
BMI=body mass index

Supplementary Table 3: Baseline characteristics of 121,433 participants aged 35-74 years at recruitment by fifths of type 2 diabetes GRS

	Fifths of type 2 diabetes GRS					Overall
	I	II	III	IV	V	
No. of participants	24,286	24,287	24,287	24,287	24,286	121,433
**Age, sex, ancestry and socioeconomic factors**						
Age, years	51.2 (11)	51.2 (11)	50.9 (11)	50.8 (11)	50.4 (10)	50.9 (11)
Men	7897 (33)	7836 (32)	7836 (32)	7784 (32)	7831 (32)	39,184 (32)
Indigenous American ancestry, %	63	67	68	69	69	67
Resident of Coyoacán	9772 (40)	9370 (39)	9309 (38)	9300 (38)	9157 (38)	46,908 (39)
Resident of Iztapalapa	14,514 (60)	14,917 (61)	14,978 (62)	14,987 (62)	15,129 (62)	74,525 (61)
University or college educated	4340 (18)	3968 (16)	3904 (16)	3792 (16)	3599 (15)	19,603 (16)
**Lifestyle factors**						
Current smoker	7058 (29)	6931 (29)	7049 (29)	6953 (29)	7006 (29)	34,997 (29)
Current alcohol drinker	16,798 (69)	16,700 (69)	16,595 (68)	16,218 (67)	16,057 (66)	82,368 (68)
Physical activity 1+ times/week	5801 (24)	5474 (23)	5371 (22)	5307 (22)	5314 (22)	27,267 (22)
**Anthropometric measures and blood pressure**						
Height, cm	156 (9)	156 (9)	156 (9)	156 (9)	156 (9)	156 (9)
Weight, kg	71.6 (13.4)	71.3 (13.2)	70.8 (12.9)	70.7 (12.9)	69.9 (12.6)	70.8 (13.0)
BMI, kg/m^2	29.2 (5.0)	29.3 (4.9)	29.1 (4.8)	29.1 (4.8)	28.8 (4.7)	29.1 (4.8)
Waist circumference, cm	94 (12)	95 (11)	94 (11)	94 (11)	94 (11)	94 (11)
Waist-to-hip ratio	0.90 (0.07)	0.90 (0.07)	0.90 (0.07)	0.90 (0.07)	0.90 (0.07)	0.90 (0.07)
Blood pressure, mmHg						
Systolic	126 (16)	127 (16)	126 (16)	127 (16)	127 (16)	127 (16)
Diastolic	83 (10)	83 (10)	83 (10)	83 (10)	83 (10)	83 (10)
**Blood-based biomarkers**						
HbA1c, %	5.7 (1.2)	5.9 (1.4)	6.0 (1.7)	6.2 (1.8)	6.6 (2.1)	6.1 (1.7)
Triglycerides, mmol/L	1.5 (0.6)	1.6 (0.7)	1.6 (0.7)	1.6 (0.7)	1.6 (0.7)	1.6 (0.7)
LDL-cholesterol, mmol/L	1.7 (0.5)	1.7 (0.5)	1.7 (0.5)	1.7 (0.5)	1.7 (0.5)	1.7 (0.5)
Clinical LDL-cholesterol, mmol/L	2.5 (0.8)	2.5 (0.8)	2.5 (0.8)	2.5 (0.8)	2.5 (0.8)	2.5 (0.8)
HDL-cholesterol, mmol/L	1.0 (0.2)	1.0 (0.2)	1.0 (0.2)	1.0 (0.2)	1.0 (0.2)	1.0 (0.2)
Total cholesterol, mmol/L	4.3 (1.0)	4.3 (1.0)	4.3 (1.0)	4.3 (1.0)	4.3 (1.0)	4.3 (1.0)
Apolipoprotein B, g/L	0.9 (0.2)	0.9 (0.2)	0.9 (0.2)	0.9 (0.2)	0.9 (0.2)	0.9 (0.2)
Apolipoprotein A1, g/L	1.2 (0.2)	1.2 (0.2)	1.2 (0.2)	1.2 (0.2)	1.2 (0.2)	1.2 (0.2)
**Medical history**						
Type 2 diabetes						
Previously diagnosed	1371 (6)	2244 (9)	2868 (12)	3620 (15)	5330 (22)	15,433 (13)
Undiagnosed	637 (3)	924 (3)	1201 (5)	1383 (6)	1793 (7)	5938 (5)
Total	2008 (8)	3168 (13)	4069 (17)	5003 (21)	7123 (29)	21,371 (18)
Ischaemic heart disease	317 (1)	301 (1)	264 (1)	267 (1)	356 (1)	1505 (1)
Stroke	199 (1)	212 (1)	199 (1)	231 (1)	215 (1)	1056 (1)
Chronic kidney disease	203 (1)	185 (1)	197 (1)	176 (1)	226 (1)	987 (1)
Cirrhosis	30 (<0.5)	34 (<0.5)	24 (<0.5)	34 (<0.5)	38 (<0.5)	160 (<0.5)
Cancer	287 (1)	297 (1)	254 (1)	270 (1)	251 (1)	1359 (1)
Emphysema	62 (<0.5)	58 (<0.5)	49 (<0.5)	47 (<0.5)	41 (<0.5)	257 (<0.5)
**Long-term medication use**						
Any diabetes medication	1061 (4)	1738 (7)	2240 (9)	2912 (12)	4278 (18)	12,229 (10)
Any anti-hypertensive medication	3293 (14)	3467 (14)	3413 (14)	3463 (14)	3535 (15)	17,171 (14)
Any lipid-lowering medication	130 (1)	112 (<0.5)	136 (1)	130 (1)	151 (1)	659 (1)
Any anti-thrombotic medication	720 (3)	693 (3)	642 (3)	596 (2)	630 (3)	3281 (3)

Mean (SD) or n (%)
BMI=body mass index; GRS=genetic risk score

Supplementary Table 4: Associations of type 2 diabetes with risk of mortality using robust Mendelian randomisation methods based on summary-level data

Cause of death	Mortality RR (95% CI) per trebling in genetically-predicted odds of T2D			
	IVW MR*	MR-Egger†	Weighted median‡	MR-PRESSO§
Vascular	1.30 (1.22-1.38)	1.20 (1.08-1.34)	1.30 (1.16-1.46)	1.35 (1.26-1.44)
Infectious	1.19 (1.11-1.27)	1.15 (1.02-1.31)	1.22 (1.06-1.40)	1.21 (1.12-1.30)
Renal	2.15 (1.97-2.34)	1.79 (1.53-2.09)	2.09 (1.79-2.43)	2.31 (2.11-2.54)
Cancer	1.01 (0.94-1.09)	1.01 (0.88-1.15)	1.02 (0.88-1.19)	1.02 (0.94-1.10)
Cirrhosis	0.93 (0.82-1.04)	0.75 (0.61-0.92)	0.80 (0.64-1.00)	0.91 (0.80-1.03)
COPD	0.94 (0.78-1.14)	0.70 (0.49-1.00)	0.96 (0.65-1.41)	0.94 (0.76-1.16)
Acute diabetic	2.12 (1.84-2.45)	2.09 (1.61-2.71)	2.33 (1.74-3.11)	2.29 (1.95-2.68)
Ill-defined, other or external	1.11 (1.01-1.22)	0.97 (0.81-1.15)	1.01 (0.84-1.20)	1.12 (1.01-1.25)
All-cause	1.28 (1.23-1.32)	1.17 (1.10-1.24)	1.22 (1.14-1.30)	1.30 (1.26-1.35)

Mortality rate ratios (RRs) are per trebling in the genetically-predicted odds of type 2 diabetes (T2D) using various 'two sample' robust Mendelian randomisation methods based on summary-level data. SNP-T2D effect estimates and their standard errors are derived from Type 2 Diabetes Global Genomics Initiative multi-ancestry GWAS and SNP-mortality effect estimates and their standard errors are derived from the Mexico City Prospective Study (adjusting for age, sex and the first 7 genetic principal components). For all approaches, the SNPs were aligned so that the SNP-T2D associations were strictly positive.

*Inverse variance weighted (IVW) Mendelian randomisation (MR) assumes all SNPs are valid instrumental variables and that none demonstrates horizontal pleiotropy.

†MR-Egger provides a robust causal estimate in the presence of directional pleiotropy, assuming it is independent of the association of each SNP with the exposure. An intercept statistically significantly different to 1.00 suggests the presence of directional pleiotropy. For each RR, the intercept term was non-significant at the Bonferroni-corrected significance level of p<0.006 (i.e., 0.05/9) with the exception of death from all causes (p=0.001; intercept=1.004 [95% CI 1.002-1.006]).

‡Weighted median MR provides a robust causal estimate if at least 50% of the weight in the analyses is derived from SNPs that are valid instrumental variables with no pleiotropic effects.

§Mendelian Randomisation Pleiotropy RESidual Sum and Outlier (MR-PRESSO) identifies SNPs with horizontal pleiotropic effects based on their contributions to heterogeneity and removes them from the analyses.

COPD=chronic obstructive pulmonary disease.

Supplementary Figure 1: Participant exclusions to derive genetic and observational analysis populations

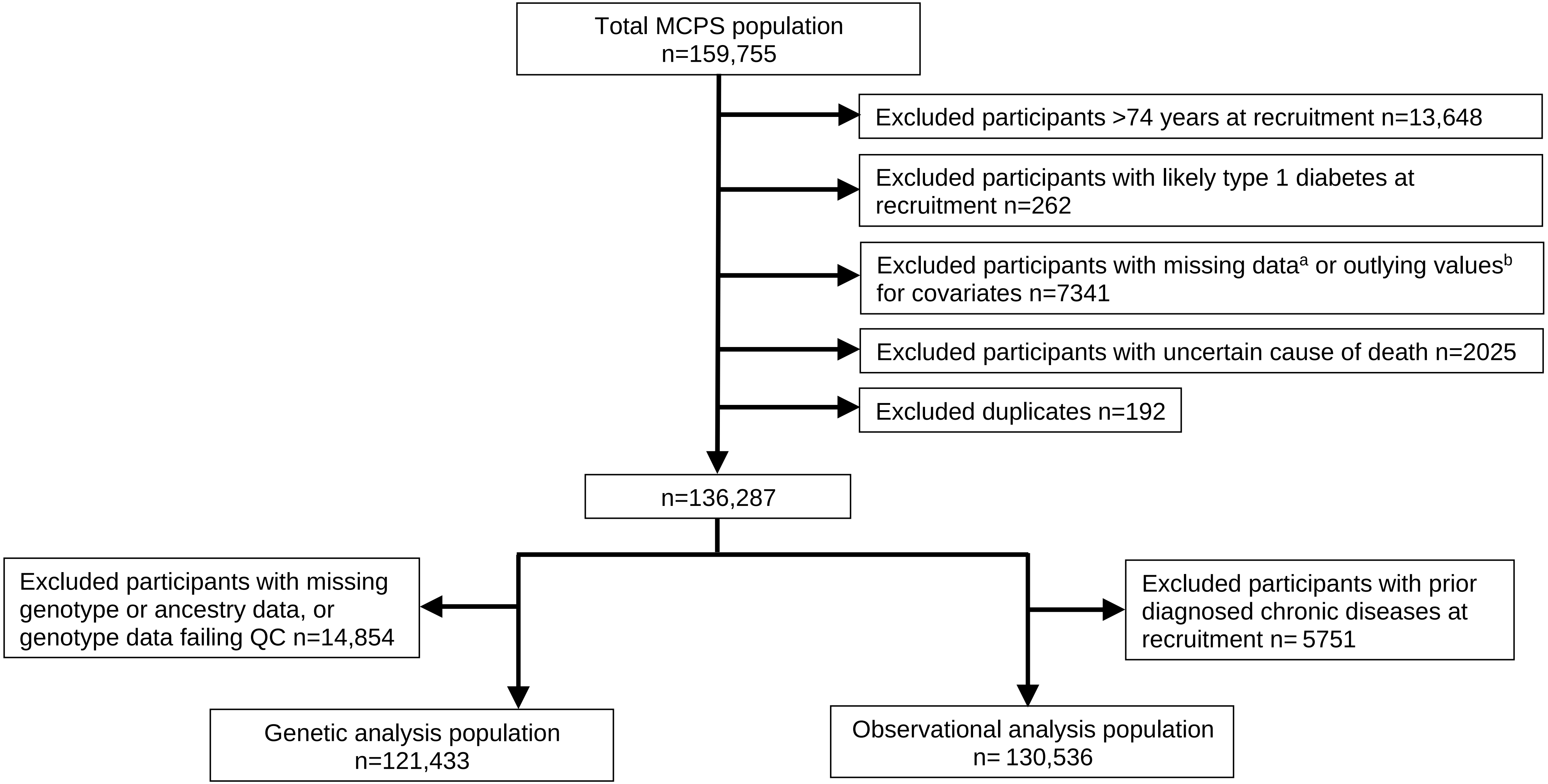

[a]Alcohol drinking, BMI, education, HbA1c, height, hip circumference, physical activity, smoking, waist circumference, weight, age at diabetes diagnosis; n=7088.
[b]Height: <140 cm or >200 cm in men, <120 cm or >180 cm in women; weight: <40 kg or >250 kg in men, <35 kg or >250 kg in women; BMI: <15 kg/m^2 or >50 kg/m^2; waist circumference: <60 cm or >150 cm; hip circumference: <70 cm or >150 cm in men, <70 cm or >160 cm in women; waist-hip-ratio <0.7 or >1.2; n=253.

Supplementary Figure 2: Genetic variant effect size estimates for type 2 diabetes in Mexico City Prospective Study and in Type 2 Diabetes Global Genomics Initiative multi-ancestry GWAS

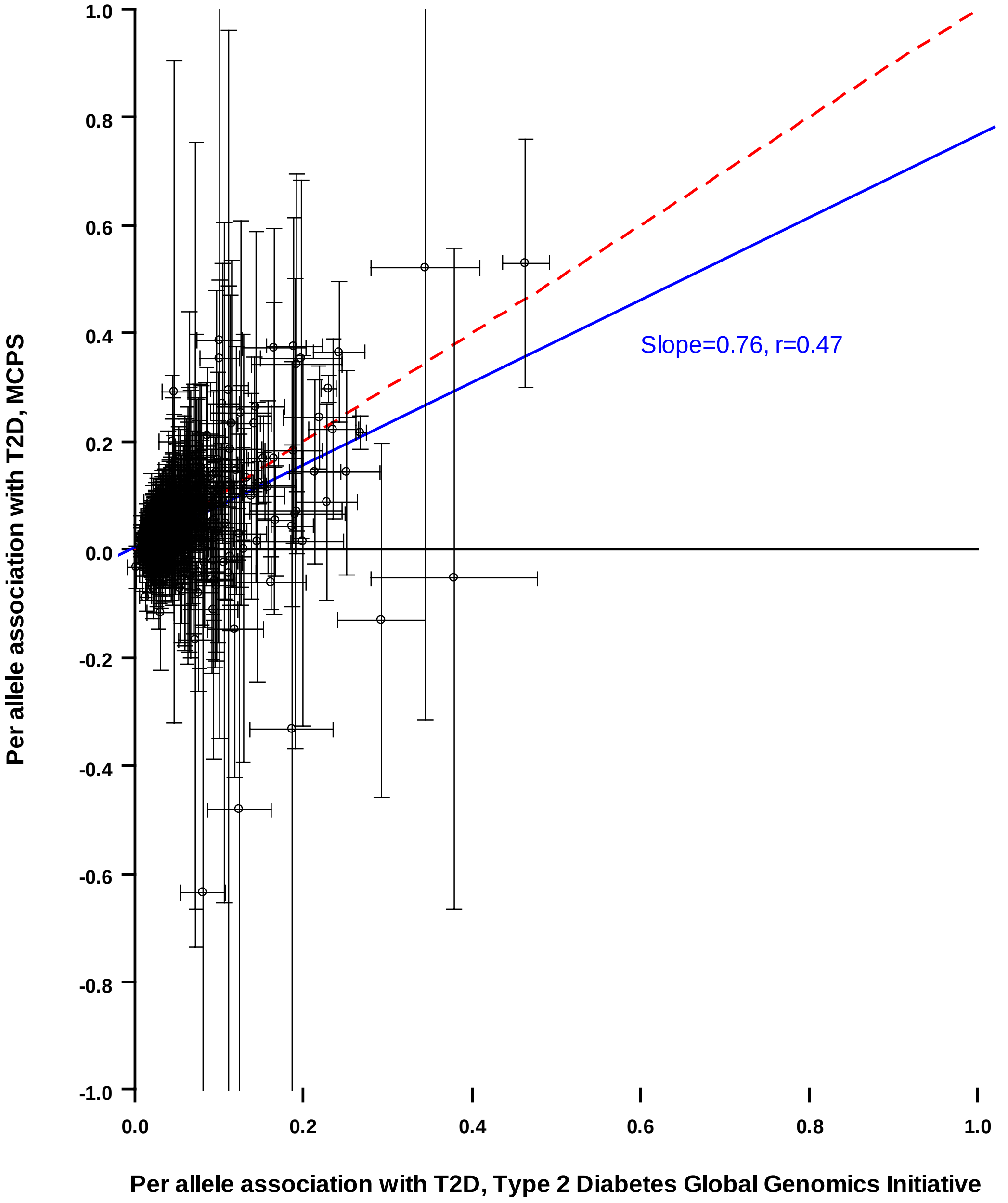

Red dashed line is line of equality. Blue line is line of best fit from a linear regression. Type 2 Diabetes Global Genomics Initiative: Nature 2024;627(8003):347-357. MCPS=Mexico City Prospective Study; r=Pearson correlation coefficient; T2D=type 2 diabetes.

Supplementary Figure 3: Association of type 2 diabetes GRS with type 2 diabetes at recruitment by sex, age and Indigenous American ancestry proportion

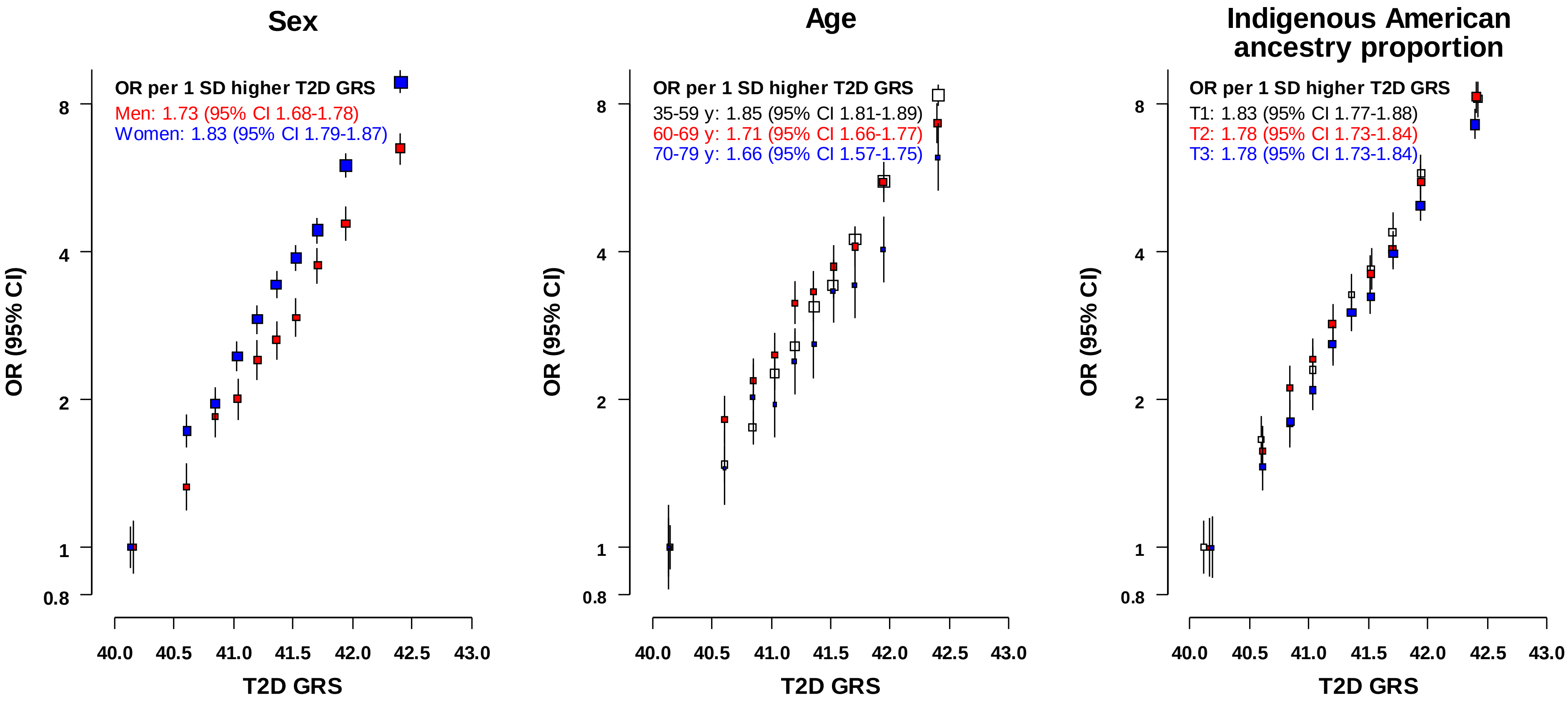

Type 2 diabetes (T2D) at recruitment refers to previously diagnosed or undiagnosed T2D. The T2D genetic risk score (GRS) represents the sum across all SNPs of their effect allele count multiplied by the log odds ratio (OR) for the association of the SNP with T2D in the T2D Global Genomics Initiative multi-ancestry meta-regression. T2D GRS SD: men=0.64 units; women=0.64 units; 35-59 y=0.64 units; 60-69 y=0.64 units; 70-79 y=0.63 units; Indigenous American ancestry proportion T1=0.67 units; Indigenous American ancestry proportion T2=0.64 units; Indigenous American ancestry proportion T3=0.60 units. T2D ORs are adjusted for age, sex and the first 7 genetic principal components, where appropriate. The size of each square is proportional to the amount of data available. Error bars represent 95% confidence intervals (CI). T=third.

Supplementary Figure 4: Association of Hispanic type 2 diabetes GRS with type 2 diabetes at recruitment

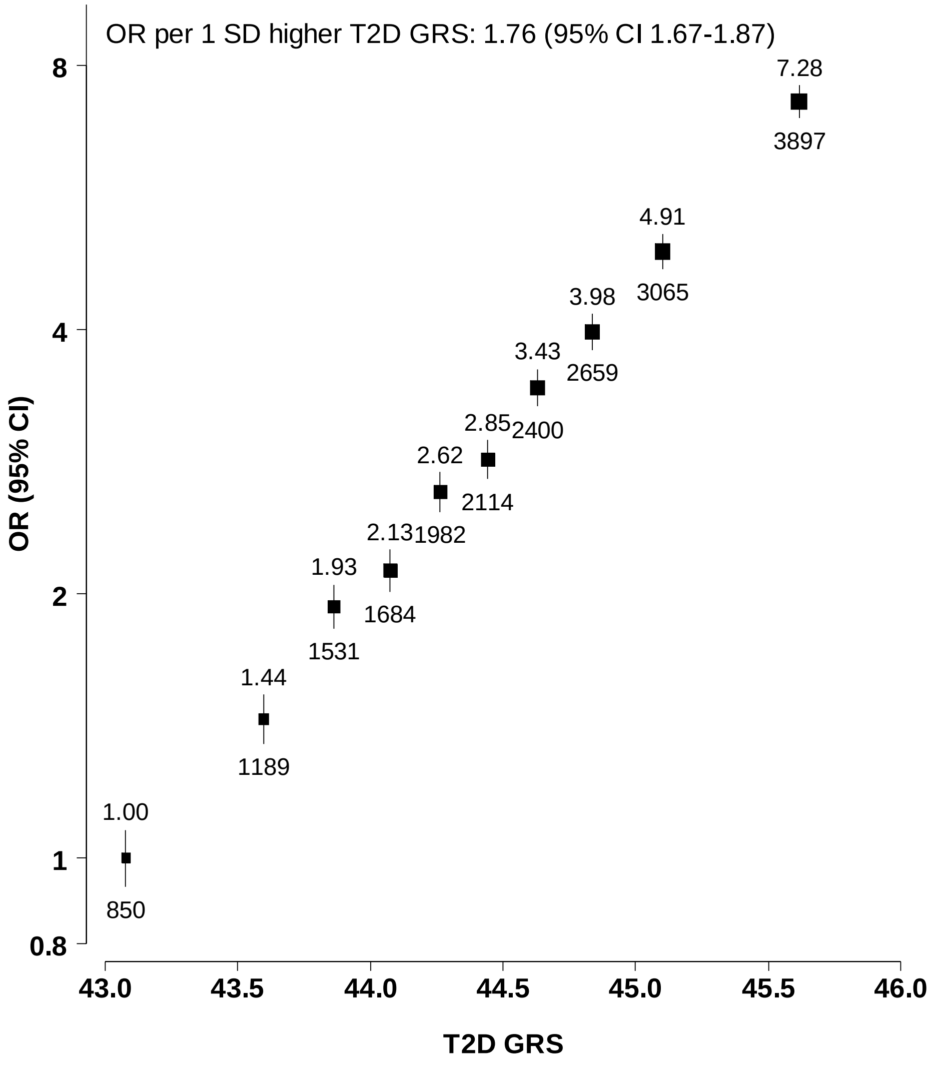

Type 2 diabetes (T2D) at recruitment refers to previously diagnosed or undiagnosed T2D. The T2D genetic risk score (GRS) represents the sum across all SNPs of their effect allele count multiplied by the log odds ratio (OR) for the association of the SNP with T2D from Hispanic ancestry groups within the T2D Global Genomics Initiative. T2D GRS SD=0.72 units. T2D ORs are adjusted for age, sex and the first 7 genetic principal components. The numbers above the squares are the ORs and the numbers below the squares are the number of participants with T2D in that group. The size of each square is proportional to the amount of data available. Error bars represent 95% confidence intervals (CI).

Supplementary Figure 5: Cause-specific mortality rate ratios per 1-unit higher log-odds of genetically-predicted type 2 diabetes at ages 35-74 years

Cause of death	No. of deaths	RR (95% CI)
Vascular		
Cardiac	2287	1.27 (1.14, 1.42)
Cerebrovascular	695	1.24 (1.01, 1.51)
Other vascular	244	1.55 (1.11, 2.17)
**Any**	**3226**	**1.28 (1.17, 1.41)**
Infectious		
Urinary tract	203	1.54 (1.06, 2.23)
Skin, bone and connective tissue	118	1.04 (0.64, 1.70)
Septicaemia	186	1.74 (1.18, 2.57)
Gastrointestinal	179	1.17 (0.79, 1.74)
Respiratory	1490	1.11 (0.96, 1.27)
Other	261	1.16 (0.83, 1.60)
**Any**	**2437**	**1.19 (1.06, 1.32)**
**Renal**	**1696**	**2.13 (1.87, 2.42)**
Cancer		
Lung	141	1.02 (0.65, 1.58)
Liver, gallbladder or bile duct	234	0.95 (0.67, 1.34)
Urinary tract	112	1.26 (0.77, 2.07)
Colorectal	155	0.88 (0.58, 1.35)
Oesophageal/stomach	215	0.99 (0.69, 1.43)
Cervix	112	0.97 (0.59, 1.61)
Female breast	227	1.01 (0.71, 1.43)
Haematological	182	1.04 (0.71, 1.54)
Prostate	67	1.04 (0.55, 1.97)
Endometrial	35	0.85 (0.35, 2.06)
Pancreas	103	0.92 (0.55, 1.55)
Ovary	107	1.03 (0.61, 1.71)
Upper aerodigestive	35	0.98 (0.40, 2.38)
Other defined site	193	0.97 (0.66, 1.42)
Ill-defined	98	1.27 (0.75, 2.16)
**Any**	**2016**	**1.00 (0.89, 1.13)**
**Cirrhosis**	**895**	**0.91 (0.76, 1.09)**
**COPD**	**287**	**0.92 (0.67, 1.25)**
**Acute diabetic**	**509**	**2.11 (1.67, 2.66)**
Ill-defined, other or external		
Ill-defined	234	1.24 (0.88, 1.75)
Other	656	1.13 (0.92, 1.39)
External	337	0.99 (0.74, 1.32)
**Any**	**1227**	**1.11 (0.95, 1.29)**
**All-cause**	**12293**	**1.26 (1.21, 1.33)**

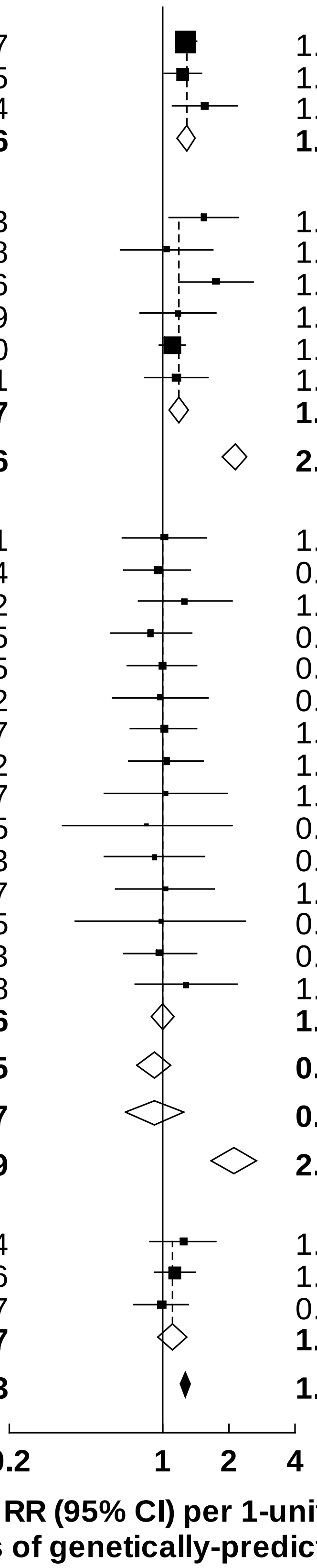

Mortality rate ratios (RRs) are per 1-unit higher log-odds of genetically-predicted risk of type 2 diabetes (T2D) and are stratified by age-at-risk and sex and adjusted for the first 7 genetic principal components. The size of each square is inversely proportional to the variance of the log RR. Horizontal lines represent 95% confidence intervals (CI). COPD=chronic obstructive pulmonary disease.

Supplementary Figure 6: Observational associations of previously diagnosed and undiagnosed type 2 diabetes with cause-specific mortality at ages 35-74 years

Cause of death	Type 2 diabetes	No. of deaths	RR (95% CI)
Vascular	Undiagnosed	321	2.48 (2.20, 2.80)
	Diagnosed	1117	3.68 (3.40, 3.98)
Infectious	Undiagnosed	252	2.54 (2.22, 2.91)
	Diagnosed	782	4.12 (3.76, 4.52)
Renal	Undiagnosed	204	11.1 (9.29, 13.3)
	Diagnosed	1180	27.3 (24.0, 31.2)
Cancer	Undiagnosed	114	1.04 (0.86, 1.26)
	Diagnosed	295	1.16 (1.02, 1.32)
Cirrhosis	Undiagnosed	59	1.17 (0.89, 1.53)
	Diagnosed	157	1.41 (1.18, 1.69)
COPD	Undiagnosed	22	1.20 (0.77, 1.87)
	Diagnosed	53	1.19 (0.87, 1.62)
Acute diabetic	Undiagnosed	68	8.87 (6.55, 12.0)
	Diagnosed	325	19.1 (15.4, 23.8)
Ill-defined, other or external	Undiagnosed	86	1.42 (1.13, 1.77)
	Diagnosed	291	2.05 (1.78, 2.35)
All-cause	Undiagnosed	1126	2.32 (2.18, 2.47)
	Diagnosed	4200	3.86 (3.71, 4.02)

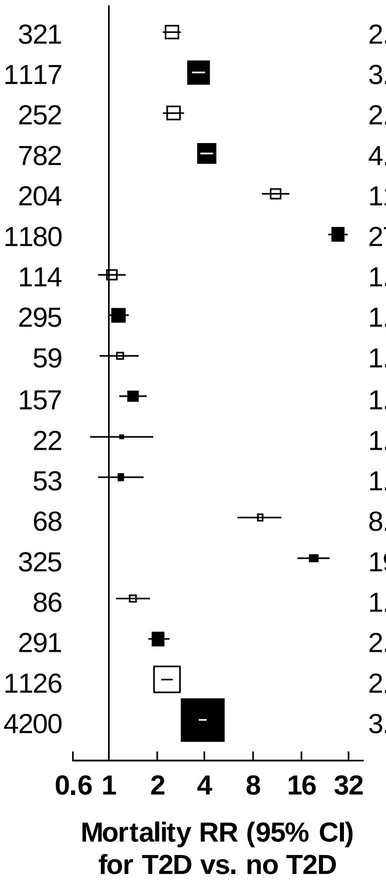

Mortality rate ratios (RRs) are stratified by age-at-risk and sex and adjusted for district, educational level, smoking status, alcohol drinking, height, weight, waist circumference and hip circumference. The size of each square is inversely proportional to the variance of the log RR. Horizontal lines represent 95% confidence intervals. COPD=chronic obstructive pulmonary disease.

Supplementary Figure 7: Genetic and observational associations of type 2 diabetes with vascular mortality at ages 35-74 years

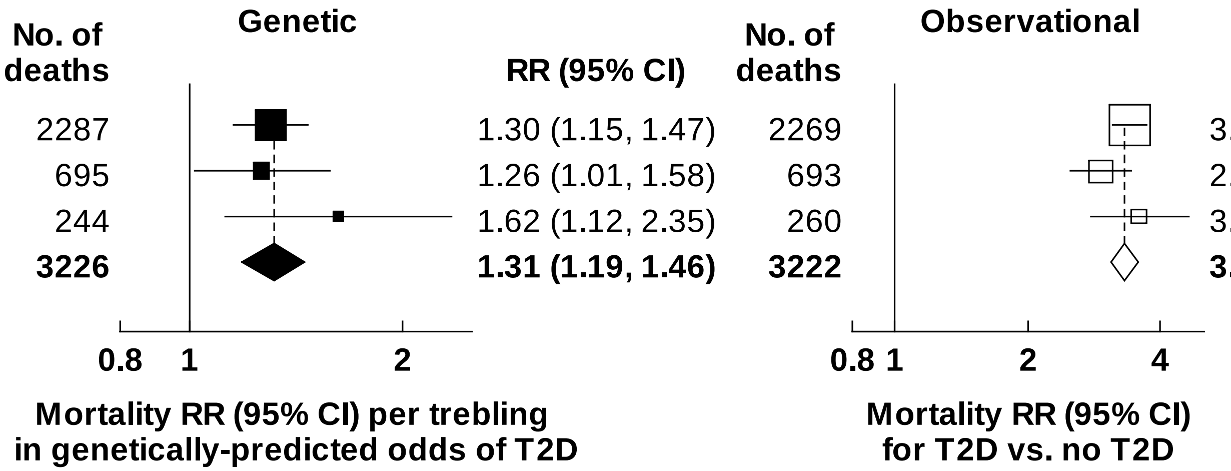

Cause of death	Genetic: No. of deaths	Genetic: RR (95% CI)	Observational: No. of deaths	Observational: RR (95% CI)
Cardiac	2287	1.30 (1.15, 1.47)	2269	3.41 (3.12, 3.72)
Cerebrovascular	695	1.26 (1.01, 1.58)	693	2.93 (2.50, 3.43)
Other vascular	244	1.62 (1.12, 2.35)	260	3.57 (2.77, 4.62)
**Any**	**3226**	**1.31 (1.19, 1.46)**	**3222**	**3.31 (3.08, 3.56)**

Genetic mortality rate ratios (RRs) are per trebling in the genetically-predicted odds of type 2 diabetes (T2D) and are stratified by age-at-risk and sex and adjusted for the first 7 genetic principal components. T2D in observational associations refers to previously diagnosed or undiagnosed T2D. Observational mortality RRs are stratified by age-at-risk and sex and adjusted for district, educational level, smoking status, alcohol drinking, height, weight, waist circumference and hip circumference. The size of each square is inversely proportional to the variance of the log RR. Horizontal lines represent 95% confidence intervals (CI).

Supplementary Figure 8: Genetic and observational associations of type 2 diabetes with infectious mortality at ages 35-74 years

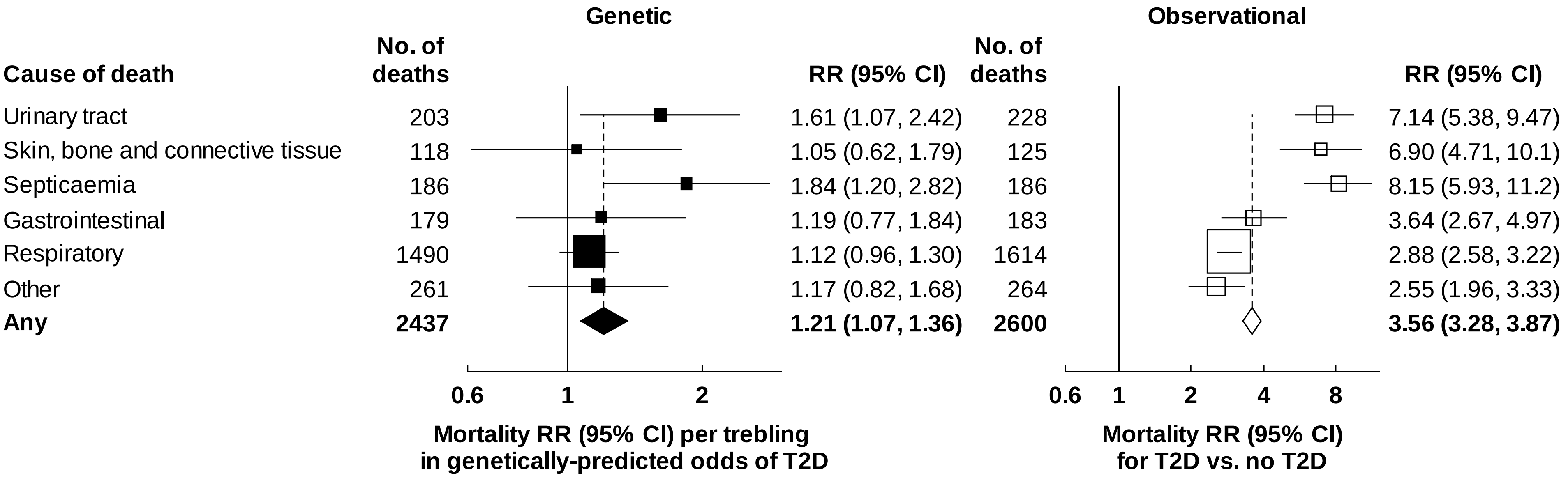

Genetic mortality rate ratios (RRs) are per trebling in the genetically-predicted odds of type 2 diabetes (T2D) and are stratified by age-at-risk and sex and adjusted for the first 7 genetic principal components. T2D in observational associations refers to previously diagnosed or undiagnosed T2D. Observational mortality RRs are stratified by age-at-risk and sex and adjusted for district, educational level, smoking status, alcohol drinking, height, weight, waist circumference and hip circumference. The size of each square is inversely proportional to the variance of the log RR. Horizontal lines represent 95% confidence intervals (CI).

Supplementary Figure 9: Genetic and observational associations of type 2 diabetes with cancer mortality at ages 35-74 years

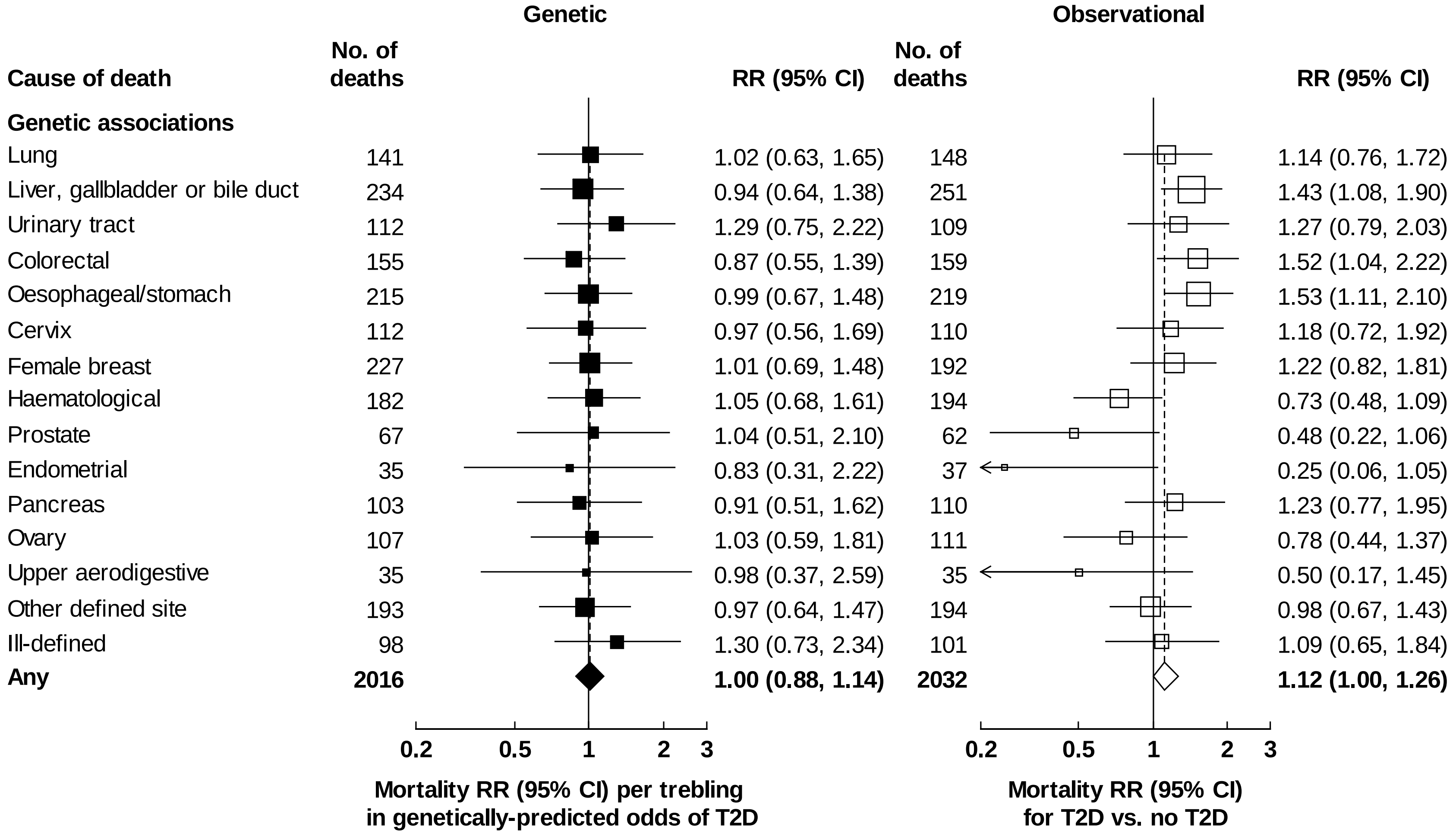

Genetic mortality rate ratios (RRs) are per trebling in the genetically-predicted odds of type 2 diabetes (T2D) and are stratified by age-at-risk and sex and adjusted for the first 7 genetic principal components. T2D in observational associations refers to previously diagnosed or undiagnosed T2D. Observational mortality RRs are stratified by age-at-risk and sex and adjusted for district, educational level, smoking status, alcohol drinking, height, weight, waist circumference and hip circumference. The size of each square is inversely proportional to the variance of the log RR. Horizontal lines represent 95% confidence intervals (CI).

Supplementary Figure 10: Genetic and observational associations of type 2 diabetes with ill-defined, other medical or external mortality at ages 35-74 years

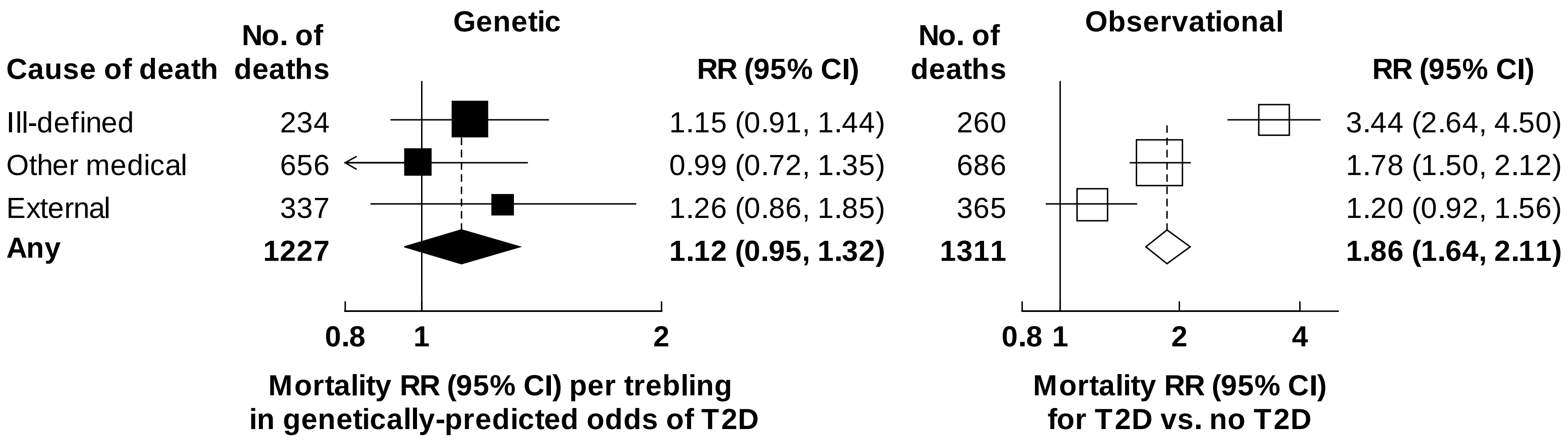

Genetic mortality rate ratios (RRs) are per trebling in the genetically-predicted odds of type 2 diabetes (T2D) and are stratified by age-at-risk and sex and adjusted for the first 7 genetic principal components. T2D in observational associations refers to previously diagnosed or undiagnosed T2D. Observational mortality RRs are stratified by age-at-risk and sex and adjusted for district, educational level, smoking status, alcohol drinking, height, weight, waist circumference and hip circumference. The size of each square is inversely proportional to the variance of the log RR. Horizontal lines represent 95% confidence intervals (CI).

Supplementary Figure 11: Relevance of pathway specific type 2 diabetes GRS to cause-specific mortality at ages 35-74 years

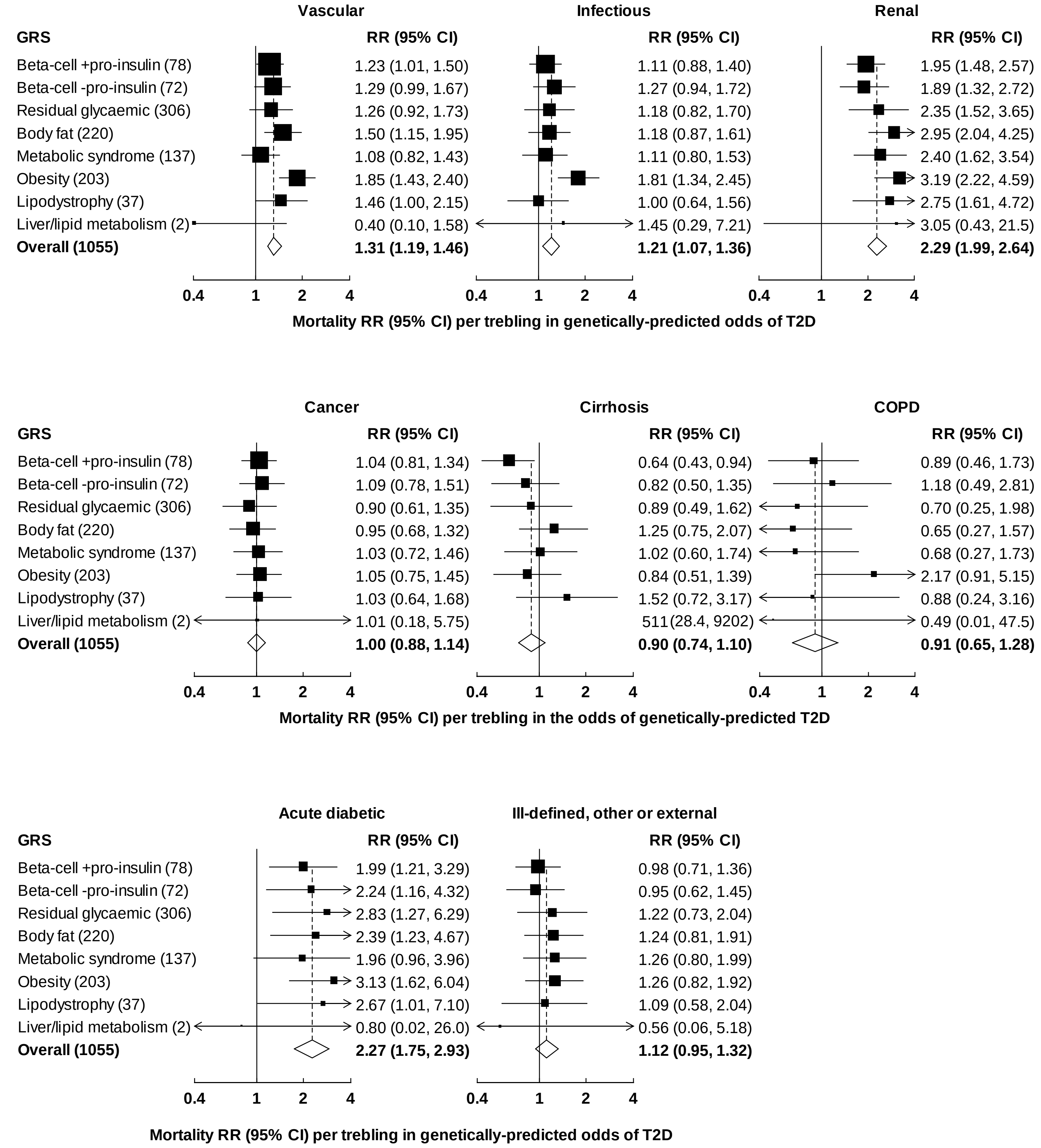

Mortality rate ratios (RRs) are per trebling in the genetically-predicted odds of type 2 diabetes (T2D) and are stratified by age-at-risk and sex and adjusted for the first 7 genetic principal components. The size of each square is inversely proportional to the variance of the log RR. Horizontal lines represent 95% confidence intervals (CI). COPD=chronic obstructive pulmonary disease; GRS=genetic risk score.

Supplementary Figure 12: Genetic associations of type 2 diabetes with cause-specific mortality, by age-at-risk

Cause of death	No. of deaths	RR (95% CI)
Vascular		
35-59 y	751	1.43 (1.16, 1.76)
60-69 y	1427	1.36 (1.17, 1.59)
70-74 y	1048	1.18 (0.99, 1.41)
Infectious		
35-59 y	684	1.27 (1.02, 1.59)
60-69 y	1070	1.23 (1.03, 1.47)
70-74 y	683	1.11 (0.89, 1.39)
Renal		
35-59 y	510	3.23 (2.50, 4.18)
60-69 y	774	2.12 (1.71, 2.61)
70-74 y	412	1.74 (1.30, 2.33)
Cancer		
35-59 y	679	1.04 (0.83, 1.29)
60-69 y	822	0.96 (0.79, 1.18)
70-74 y	515	1.03 (0.79, 1.33)
Cirrhosis		
35-59 y	351	0.91 (0.66, 1.24)
60-69 y	381	0.89 (0.66, 1.20)
70-74 y	163	0.93 (0.58, 1.47)
COPD		
35-59 y	49	0.61 (0.27, 1.40)
60-69 y	116	1.06 (0.62, 1.81)
70-74 y	122	0.93 (0.55, 1.56)
Acute diabetic		
35-59 y	144	2.32 (1.43, 3.77)
60-69 y	232	2.18 (1.49, 3.19)
70-74 y	133	2.38 (1.44, 3.93)
Ill-defined, other or external		
35-59 y	467	1.17 (0.89, 1.53)
60-69 y	470	1.11 (0.85, 1.45)
70-74 y	290	1.06 (0.75, 1.50)
All-cause		
35-59 y	3635	1.38 (1.26, 1.53)
60-69 y	5292	1.30 (1.20, 1.41)
70-74 y	3366	1.19 (1.08, 1.32)

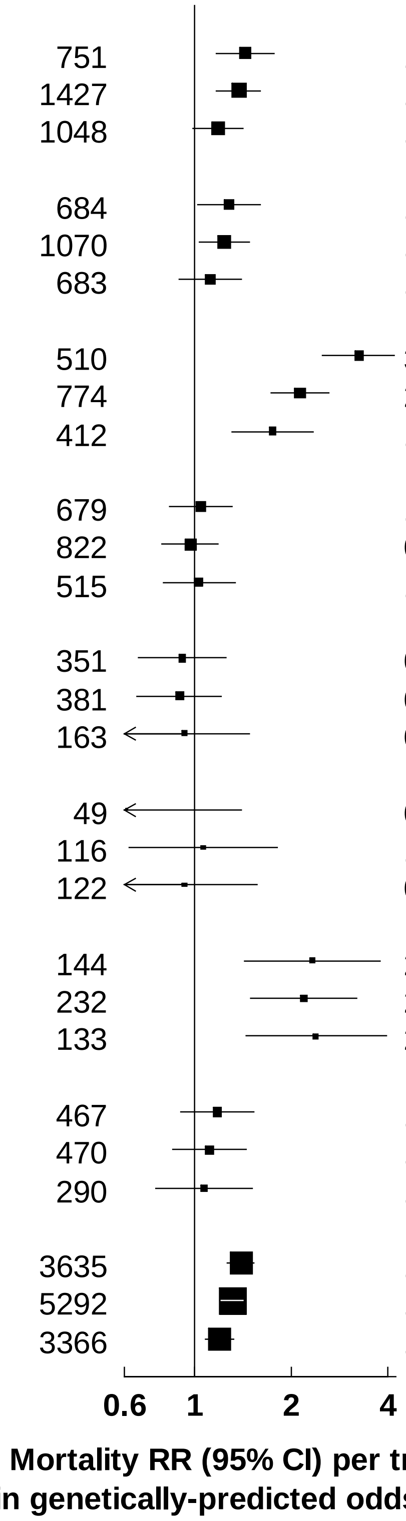

Mortality rate ratios (RRs) are per trebling in the genetically-predicted odds of type 2 diabetes (T2D) and are stratified by age-at-risk and sex and adjusted for the first 7 genetic principal components. The size of each square is inversely proportional to the variance of the log RR. Horizontal lines represent 95% confidence intervals (CI). COPD=chronic obstructive pulmonary disease.

Supplementary Figure 13: Genetic and observational associations of type 2 diabetes with cause-specific mortality at ages 75-84 years

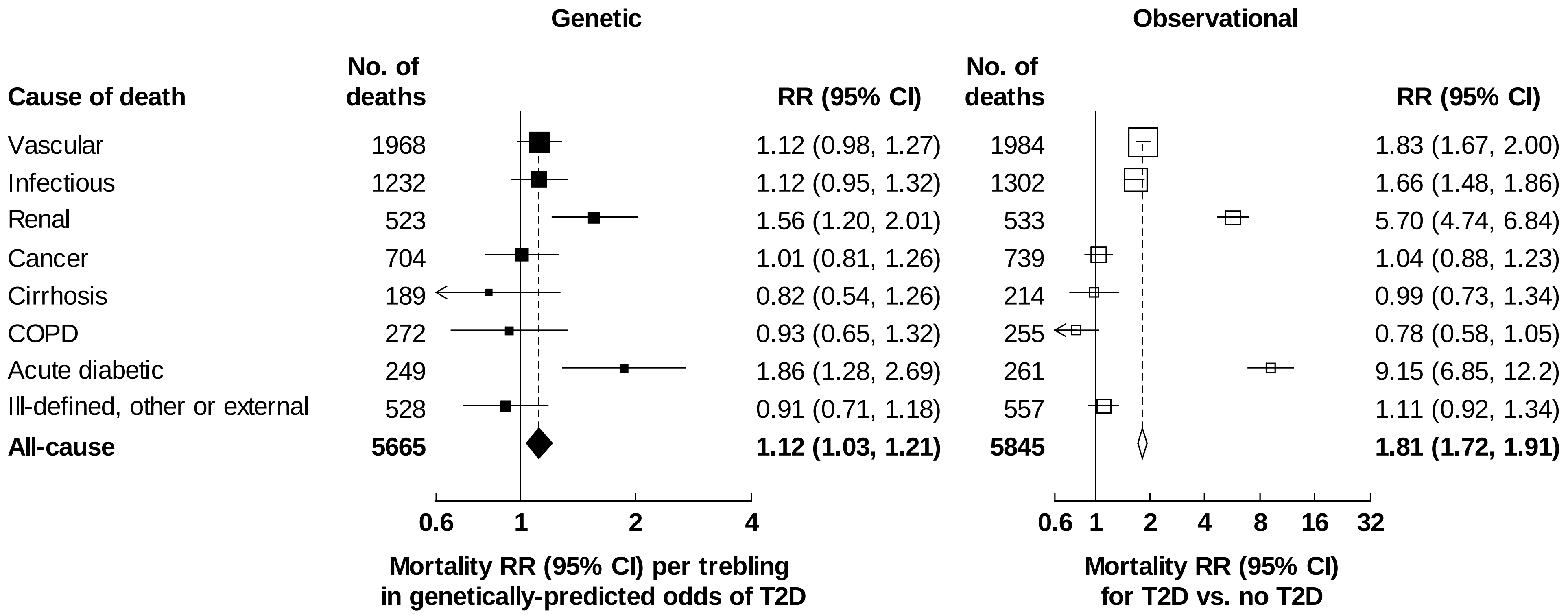

Genetic mortality rate ratios (RRs) are per trebling in the genetically-predicted odds of type 2 diabetes (T2D) and are stratified by age-at-risk and sex and adjusted for the first 7 genetic principal components. T2D in observational associations refers to previously diagnosed or undiagnosed T2D. Observational mortality RRs are stratified by age-at-risk and sex and adjusted for district, educational level, smoking status, alcohol drinking, height, weight, waist circumference and hip circumference. The size of each square is inversely proportional to the variance of the log RR. Horizontal lines represent 95% confidence intervals (CI). COPD=chronic obstructive pulmonary disease.

Supplementary Figure 14: Genetic and observational associations of type 2 diabetes with cause-specific mortality at ages 35-84 years

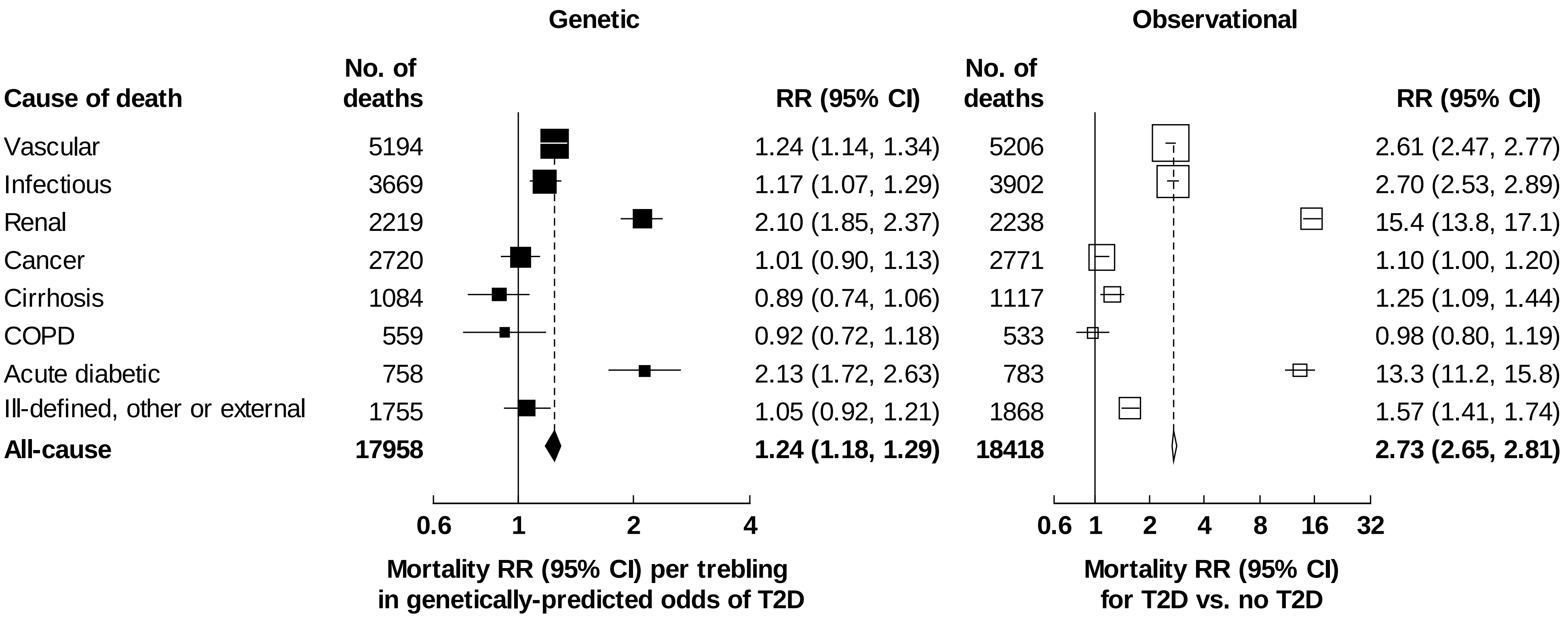

Genetic mortality rate ratios (RRs) are per trebling in the genetically-predicted odds of type 2 diabetes (T2D) and are stratified by age-at-risk and sex and adjusted for the first 7 genetic principal components. T2D in observational associations refers to previously diagnosed or undiagnosed T2D. Observational mortality RRs are stratified by age-at-risk and sex and adjusted for district, educational level, smoking status, alcohol drinking, height, weight, waist circumference and hip circumference. The size of each square is inversely proportional to the variance of the log RR. Horizontal lines represent 95% confidence intervals (CI). COPD=chronic obstructive pulmonary disease.

Supplementary Figure 15: Genetic associations of type 2 diabetes with cause-specific mortality at ages 35-74 years, by sex

Cause of death	No. of deaths	RR (95% CI)
Vascular		
Men	1515	1.32 (1.11, 1.57)
Women	1711	1.32 (1.16, 1.51)
Infectious		
Men	1043	1.12 (0.91, 1.38)
Women	1394	1.27 (1.10, 1.47)
Renal		
Men	703	2.30 (1.78, 2.97)
Women	993	2.31 (1.94, 2.75)
Cancer		
Men	656	1.14 (0.88, 1.48)
Women	1360	0.95 (0.82, 1.10)
Cirrhosis		
Men	518	0.84 (0.62, 1.13)
Women	377	0.98 (0.74, 1.30)
COPD		
Men	133	0.89 (0.50, 1.58)
Women	154	0.93 (0.60, 1.43)
Acute diabetic		
Men	224	2.37 (1.51, 3.71)
Women	285	2.23 (1.62, 3.07)
Ill-defined, other or external		
Men	546	1.07 (0.81, 1.43)
Women	681	1.16 (0.94, 1.42)
All-cause		
Men	5338	1.28 (1.17, 1.41)
Women	6955	1.31 (1.23, 1.40)

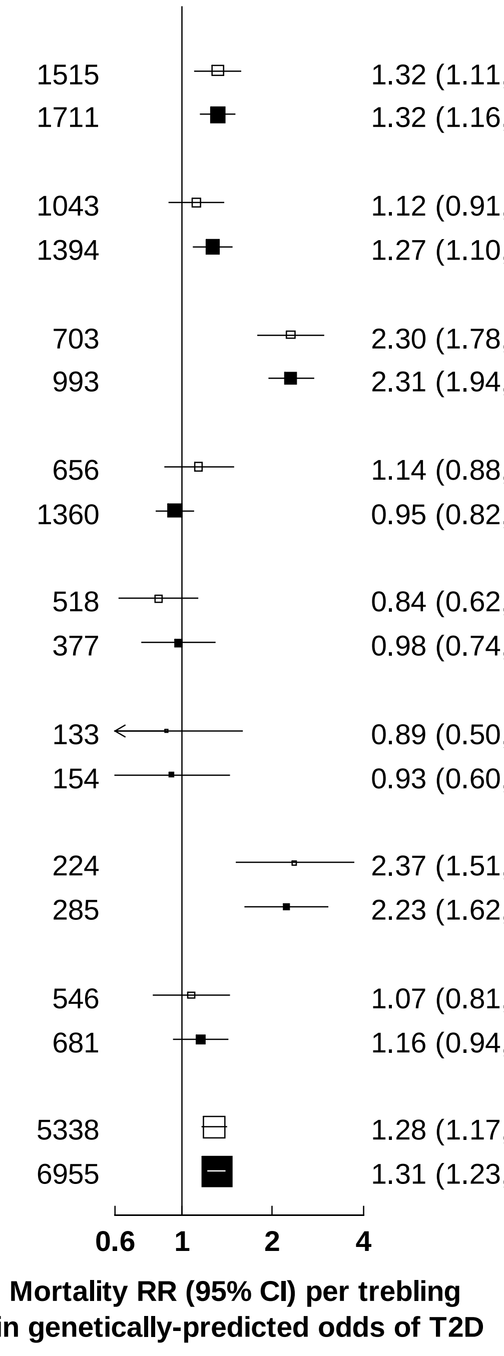

Mortality rate ratios (RRs) are per trebling in the genetically-predicted odds of type 2 diabetes (T2D) and are stratified by age-at-risk and adjusted for the first 7 genetic principal components. The size of each square is inversely proportional to the variance of the log RR. Horizontal lines represent 95% confidence intervals (CI). COPD=chronic obstructive pulmonary disease.

Supplementary Figure 16: Genetic associations of type 2 diabetes with cause-specific mortality at ages 35-74 years, by district

Cause of death	No. of deaths	RR (95% CI)
Vascular		
Coyoacán	1084	1.28 (1.09, 1.51)
Iztapalapa	2142	1.34 (1.17, 1.52)
Infectious		
Coyoacán	752	1.28 (1.05, 1.56)
Iztapalapa	1685	1.18 (1.02, 1.36)
Renal		
Coyoacán	476	2.39 (1.86, 3.07)
Iztapalapa	1220	2.27 (1.91, 2.69)
Cancer		
Coyoacán	727	0.99 (0.81, 1.22)
Iztapalapa	1289	1.01 (0.86, 1.19)
Cirrhosis		
Coyoacán	320	1.00 (0.74, 1.36)
Iztapalapa	575	0.85 (0.66, 1.10)
COPD		
Coyoacán	86	0.77 (0.43, 1.38)
Iztapalapa	201	0.99 (0.65, 1.50)
Acute diabetic		
Coyoacán	140	2.51 (1.59, 3.97)
Iztapalapa	369	2.19 (1.61, 2.99)
Ill-defined, other or external		
Coyoacán	467	1.15 (0.90, 1.48)
Iztapalapa	760	1.10 (0.88, 1.36)
All-cause		
Coyoacán	4052	1.29 (1.18, 1.40)
Iztapalapa	8241	1.30 (1.22, 1.39)

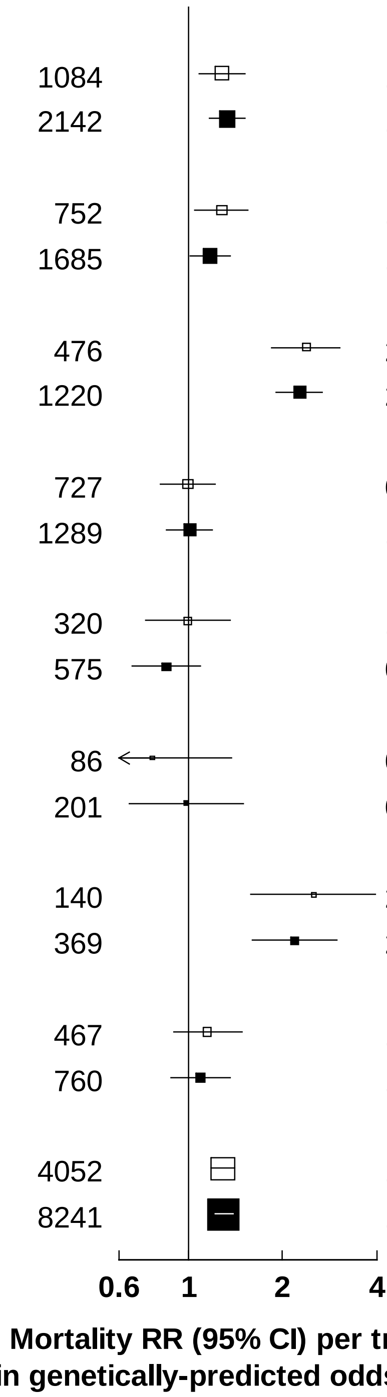

Mortality rate ratios (RRs) are per trebling in the genetically-predicted odds of type 2 diabetes (T2D) and are stratified by age-at-risk and sex and adjusted for the first 7 genetic principal components. The size of each square is inversely proportional to the variance of the log RR. Horizontal lines represent 95% confidence intervals (CI). COPD=chronic obstructive pulmonary disease.

Supplementary Figure 17: Genetic associations of type 2 diabetes with cause-specific mortality at ages 35-74 years, by relatedness

Cause of death		No. of deaths	RR (95% CI)
Vascular	All	3226	1.31 (1.19, 1.46)
	Unrelated	1772	1.30 (1.14, 1.49)
Infectious	All	2437	1.21 (1.07, 1.36)
	Unrelated	1285	1.22 (1.04, 1.44)
Renal	All	1696	2.29 (1.99, 2.64)
	Unrelated	913	2.23 (1.84, 2.70)
Cancer	All	2016	1.00 (0.88, 1.14)
	Unrelated	1128	1.05 (0.88, 1.24)
Cirrhosis	All	895	0.90 (0.74, 1.10)
	Unrelated	442	0.91 (0.69, 1.21)
COPD	All	287	0.91 (0.65, 1.28)
	Unrelated	170	0.91 (0.59, 1.42)
Acute diabetic	All	509	2.27 (1.75, 2.93)
	Unrelated	270	2.51 (1.77, 3.57)
Ill-defined, other or external	All	1227	1.12 (0.95, 1.32)
	Unrelated	663	1.17 (0.94, 1.47)
All-cause	All	12293	1.29 (1.23, 1.36)
	Unrelated	6643	1.31 (1.22, 1.41)

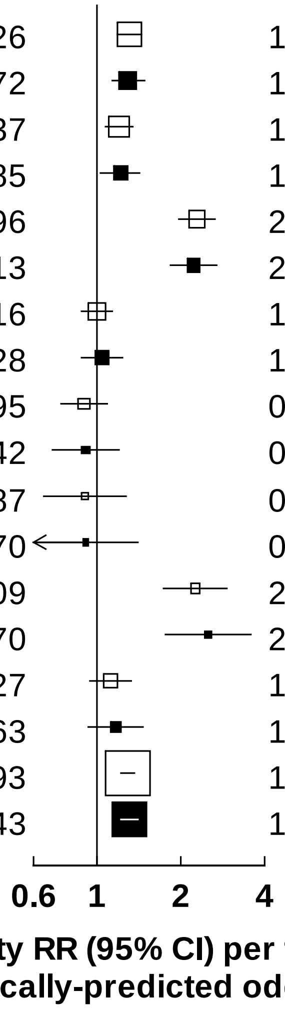

Mortality rate ratios (RRs) are per trebling in the genetically-predicted odds of type 2 diabetes (T2D) and are stratified by age-at-risk and sex and adjusted for the first 7 genetic principal components. The size of each square is inversely proportional to the variance of the log RR. Horizontal lines represent 95% confidence intervals (CI). COPD=chronic obstructive pulmonary disease.

Supplementary Figure 18: Genetic associations of type 2 diabetes with cause-specific mortality at ages 35-74 years, by Indigenous American ancestry proportion

Cause of death	No. of deaths	RR (95% CI)
**Vascular**		
Bottom third	1078	1.27 (1.08, 1.48)
Middle third	1073	1.36 (1.14, 1.63)
Top third	1075	1.33 (1.09, 1.61)
**Infectious**		
Bottom third	629	1.21 (0.98, 1.49)
Middle third	799	1.13 (0.92, 1.39)
Top third	1009	1.28 (1.05, 1.56)
**Renal**		
Bottom third	348	2.24 (1.70, 2.97)
Middle third	534	2.21 (1.71, 2.85)
Top third	814	2.40 (1.93, 2.99)
**Cancer**		
Bottom third	681	1.07 (0.87, 1.30)
Middle third	684	0.97 (0.78, 1.22)
Top third	651	0.96 (0.75, 1.23)
**Cirrhosis**		
Bottom third	199	1.02 (0.70, 1.47)
Middle third	295	1.02 (0.73, 1.44)
Top third	401	0.75 (0.55, 1.03)
**COPD**		
Bottom third	109	0.98 (0.59, 1.61)
Middle third	104	0.96 (0.53, 1.71)
Top third	74	0.72 (0.34, 1.51)
**Acute diabetic**		
Bottom third	134	1.81 (1.15, 2.83)
Middle third	161	2.48 (1.56, 3.93)
Top third	214	2.51 (1.64, 3.86)
**Ill-defined, other or external**		
Bottom third	351	1.15 (0.87, 1.52)
Middle third	428	1.02 (0.77, 1.36)
Top third	448	1.21 (0.90, 1.64)
**All-cause**		
Bottom third	3529	1.26 (1.16, 1.38)
Middle third	4078	1.28 (1.16, 1.40)
Top third	4686	1.34 (1.23, 1.47)

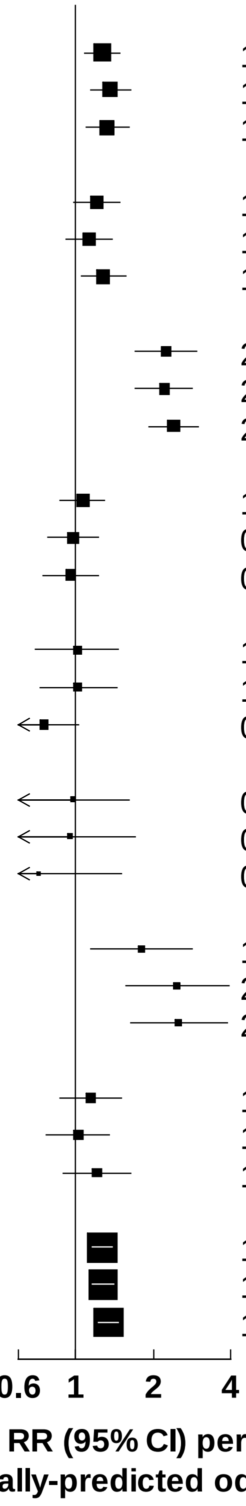

Mortality rate ratios (RRs) are per trebling in the genetically-predicted odds of type 2 diabetes (T2D) and are stratified by age-at-risk and sex and adjusted for the first 7 genetic principal components. The size of each square is inversely proportional to the variance of the log RR. Horizontal lines represent 95% confidence intervals (CI). COPD=chronic obstructive pulmonary disease.

Supplementary Figure 19: Associations of multi-ancestry and Hispanic type 2 diabetes GRSs with cause-specific mortality at ages 35-74 years

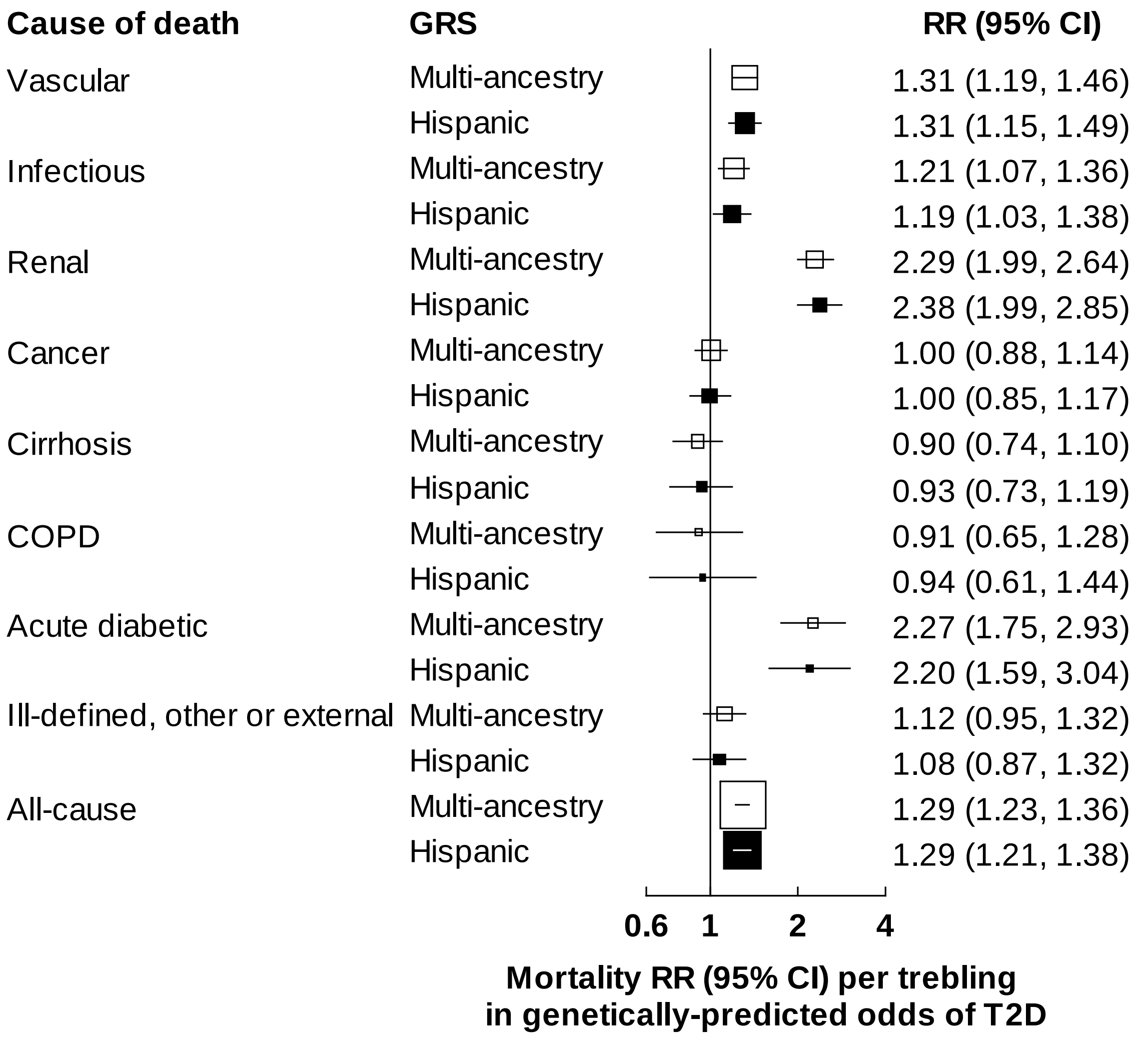

Mortality rate ratios (RRs) are per trebling in the genetically-predicted odds of type 2 diabetes (T2D) and are stratified by age-at-risk and sex and adjusted for the first 7 genetic principal components. The size of each square is inversely proportional to the variance of the log RR. Horizontal lines represent 95% confidence intervals (CI). COPD=chronic obstructive pulmonary disease.
